# Aminoglycoside antibiotic kanamycin functionalized tetraphenylethylene molecular probe for highly selective detection of bovine serum albumin protein

**DOI:** 10.1038/s41598-022-15890-4

**Published:** 2022-07-07

**Authors:** Ratan W. Jadhav, Sopan M. Wagalgave, Bajarang V. kumbhar, Rushikesh M. Khadake, Ambadas B. Rode, Sidhanath V. Bhosale, Sheshanath V. Bhosale

**Affiliations:** 1 School of Chemical Sciences, Goa University, Taleigao Plateau, Goa 403206 India; 2 Polymers and Functional Materials Division CSIR-Indian Institute of Chemical Technology, Hyderabad, Telangana 500007 India; 3 Academy of Scientific and Innovative Research (AcSIR), Ghaziabad, 201002 India; 4 Department of Biological Sciences, Sunandan Divatia School of Science, NMIMS (Deemed to be) University, Vile Parle, Mumbai, 400057 India; 5 Laboratory of Synthetic Biology, Regional Centre for Biotechnology, Faridabad, Haryana 121001 India

**Keywords:** Sensors and biosensors, Self-assembly

## Abstract

A novel tetraphenylethylene (TPE) functionalized aminoglycoside antibiotic kanamycin (TPE-kana **1**) has been successfully synthesized and characterized by means of modern analytical and spectroscopic techniques. The probe TPE-kana **1** showed strong affinity towards bovine serum albumin (BSA) compared to its other biological competitors. The recognition of BSA have been investigated employing UV–Vis absorption and fluorescence emission spectroscopy. The significant color change of TPE-kana **1** with BSA can be observed by necked eye, where the role of AIE-active TPE molecule is handle in both optical and colorimetric changes. The quenching of fluorescence of TPE-kana **1** with BSA was characterized by fluorescence spectroscopy, with 71.16% of quenching efficiency. Moreover, the Stern–Volmer quenching constant was calculated and found to be 2.46 × 10^7^ M^−1^. Probe TPE-kana **1** showed detection limit of 2.87 nM (nM) towards BSA with binding constant 7.56 × 10^7^ M. A molecular docking study is also performed to investigate the detail interactions between TPE-kana **1** with the sites of BSA via non-covalent i.e., *H*-bonding, π-cation interactions, π-donor hydrogen bonds and π-π interactions. The lowest binding energy conformation was found at − 10.42 kcal/mol.

## Introduction

In recent years, discovery of AIE-active biosensors for bioanalytes (proteins) sensing is attracted much attention. Researchers have developed several methods for sensitive and selective detection of proteins^1–9^. Most of these methods showed changes in their photophysical properties due to chemical reactions or physical interactions with proteins. Among the reported methods, the fluorescence probe methods provide an attractive advantage such as high selectivity and excellent sensitivity, rapid response and simplicity towards proteins. Bovine serum albumin (BSA) is one of the most widely investigated proteins from the serum albumin series of proteins due to its structural similarity with human serum albumin (HSA)^10^. Literature search revealed that different fluorescence reagents have been employed for the detection of BSA^2,11–16^ Most of these fluorescent materials showed aggregation caused quenching (ACQ) effect, which in turn led drastic reductions in their fluorescence emission peaks. To overcome ACQ effect, researchers utilized aggregation induced emission (AIE) materials for BSA biomolecule detection^17–21^.

The development of AIE materials to detect high sensitivity is highly challenging due to the significant role of proteins and other biomolecules in the living system^19^. Another important factor to mention, that the protein albumin exhibited structurally selective two main binding sites such as site-I and site-II, from which binding site-I mainly occurs through hydrophobic interactions, whereas binding in site-II occurs through not only hydrophobic interactions but also hydrogen bonding, and electrostatic interactions involved^2,11–21^. According to recent reports probes with high selectivity and sensitivity towards serum albumin and exhibiting specific binding at only site II^17–21^. Therefore, there is a need to develop a new, highly efficient chemosensors for the selective detection of proteins are on great demand.

On other hand, aminoglycoside antibiotics have possessed unique structurally features with number of amino and hydroxyl group. The presence of multiple amino groups on kanamycin allows it to modify through amidation reactions. The amino group at 6′ position of kanamycin is more reactive and undergoes site selective amidation, compared to other amino group due to steric hindrance factor^22^. Various amide conjugated biomaterials have been reported for different biological applications such material design, sensing of biomolecules and biomedical applications^23–26^. The molecular modelling and functionalization of aminoglycosides through covalent and non-covalent interactions have been used for the fabrications of various types of biomaterials such as hydrogels, nanoparticles, chemosensor, biointerfaces, amphiphiles and microstructures^15,27–30^. In our recent work, we have also shown that the self-assembled flower-like microstructure due to coordination of primary amino and hydroxyl group of kanamycin with Cu^2+^ ions and furthermore we have shown that flower shown to be having high surface areas, thus, used as a photocatalyst for the degradation of organic dyes^31^.

**Figure 1:**
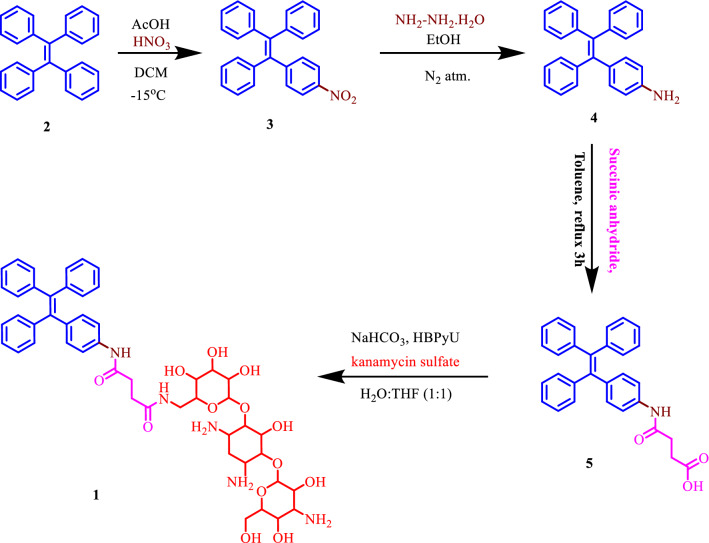
Synthetic route for TPE-kana **1.**

In this work, we described the synthesis (over four steps, Fig. 1) and photochemical properties of first ever TPE-functionalized kanamycin (kana) antibiotic (coded as: TPE-kana **1**), a strong fluorescent chromophore for selective detection of BSA via supramolecular interactions. The detection of BSA using AIE-active TPE-kana **1** in aqueous solution was found to be most powerful tool due to its easy preparation, rapid response, excellent sensitivity, high selectivity, and non-destructive nature, as the responses of TPE-kana **1** towards various other biological competitors are insignificant compared to the BSA.

## Results and discussion

### Design and synthesis of TPE-kana 1

In the present study, kanamycin, an aminoglycoside antibiotic functionalized with highly fluorescent 1,1,2,2-tetraphenylethene (TPE) molecule (emits white light), was successfully synthesized. Fluorescent TPE-functionalized kanamycin TPE-kana **1** was then used for the selective detection of BSA. The TPE-kana **1** conjugate was synthesized by a multistep reaction strategy as illustrated in Fig. 1. The (2-(4-nitrophenyl-1,1,2-triyl)tribenzene **3** was synthesized in the first step by mono-nitration of TPE **1**. The 4-(1,2,2-triphenylvinyl)aniline **4** was obtained by reducing compound **3** using hydrazine hydrate. The compound **4** was then heated with succinic anhydride in toluene yielded 4-oxo-4-((4-(1,2,2-triphenyl)phenyl)amino)butanoic acid **5**, the compound **5** was then upon amide coupling with kanamycin antibiotic resulted in the formation of target compound TPE-kana **1** (70% yield). All the compounds were confirmed by ^1^H NMR spectroscopy and the final target molecule was characterized by IR, NMR, HRMS sophisticated techniques and purity was confirmed by high-pressure liquid chromatography (HPLC), and also optical rotation as illustrated in ESI Fig. S2–S9. Typically, the strong peaks at 1660 in the IR spectrum of TPE-kana **1** attributed to amide > C=O stretching of –CONH and peaks at 1589, 1521 are attributed to –N–H bending modes of secondary amide. The ^1^H NMR spectrum of TPE-Kana **1** was recorded in deuterated methanol solvent. The chemical shift (δ) values in the region from 7.71 to 7.67 ppm and 7.29 to 7.26 ppm correspond to two H-bonded amide protons. The formation of compound TPE-kana **1** was further confirmed by the HRMS**:** (m/z) calculated 912.4025 found 912.4036 [M]^+^, 980.2810 [M + 3Na]^+^. The purity of TPE-Kana **1** was analyzed by HPLC and shown to be 95% purity of TPE-kana **1**. The specific rotation for the TPE-kana **1** was calculated using a polarimeter, the obtained specific rotation is α^23^_D_ = − 1.4929 (c 0.7, DMSO).

### Optical properties of TPE-kana 1 at different pH

The pH (4.0, 7.0 and 9.2) dependent changes of TPE-kana **1** in water were investigated using UV–Vis absorption and fluorescence spectroscopy (Fig. S10). In UV–Vis absorption spectra TPE-kana **1** showed absorption band at 310 nm, whereas there are not much differences in the absorption spectra of TPE-kana **1** at different pH. Moreover, in fluorescence emission spectra of TPE-kana **1** almost negligible emission observed at ph-4, moderate emission at pH-9.2 and the highest emission intensity was observed at pH-7. The TPE-kana **1** possess many hydroxyls (–OH) and primary amino (–NH_2_) group of kanamycin functionalized TPE as a AIE active moiety. The presence of multiple -OH and amine –NH_2_ group of kanamycin and TPE group leads to such different absorption and emission behavior of TPE-kana **1**. The multiple –OH and amine –NH_2_ group kanamycin could provide utmost opportunities for the formation of both non-covalent interactions at different pH which may leads to construction of versatile biomaterial framework. Moreover, this different behavior of TPE-kana **1** at different pH was also characterized using scanning electron microscopy (SEM) which shows almost similar mode of aggregations at pH-4 and 9.2, a globular particular aggregates like structure of TPE-kana **1** was observed. Whereas at pH-7 the TPE-kana **1** produces leaf-like structures (Fig. S11) and in pure THF solvent TPE-kana 1 self-assembled into globular structure (Fig. S12).

### AIE (aggregation induced emission) properties of TPE-kana 1

**Figure 2:**
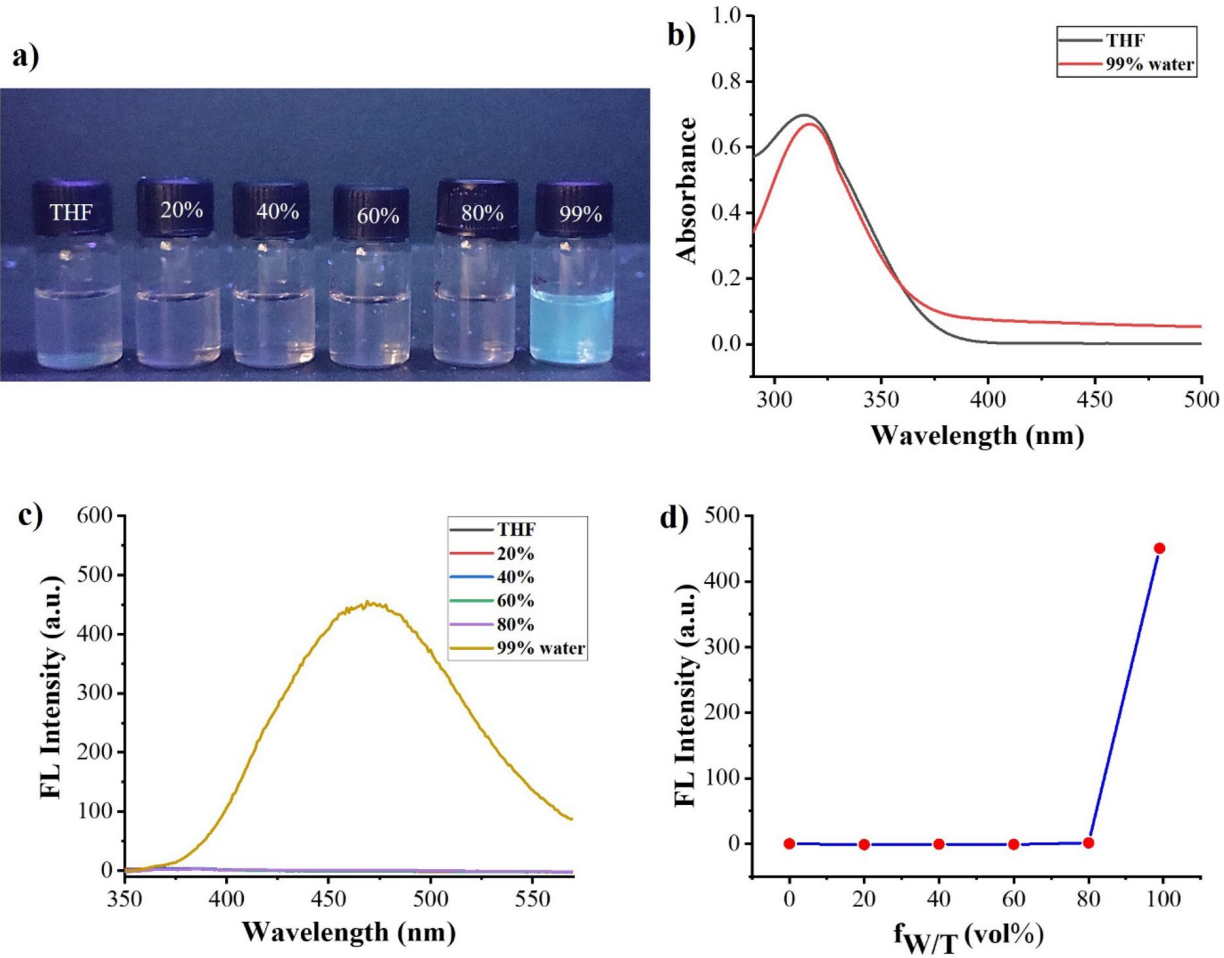
Images of TPE-kana **1** (5 × 10^−5^ M) (**a**) in THF/H_2_O mixtures with different *f*_w_ (0–99%) under 365 nm UV light. (**b**) UV–Vis absorption spectra in THF and THF/H_2_O *f*_w_ (0–99%). (**c**) Fluorescence emission spectra of the TPE-kana **1** in THF/H_2_O (*v/v* ratio) mixtures with different water fractions (λ_ex_ = 310 nm) and (**d**) plot of relative fluorescence emission intensity as a function of *f*_w_.

After successful synthesis of TPE-kana **1**, we investigate whether the kanamycin antibiotic affect the AIE behavior (restriction to intramolecular rotation or vibrational motion) of TPE-kana **1** we studied UV–Vis absorption and fluorescence emission behavior of TPE-kana **1** in different proportion of THF-water mixtures. TPE is a strong AIE active compound, exhibits AIE behavior upon aggregation, and shows strong emission of light. The naked eye detection study showed that upon increasing the water ratio up to 99% the significant increase in the fluorescence was observed under 365 nm UV-light Fig. 2a. Moreover, the AIE characteristics of TPE-kana **1** were investigated using UV − Vis absorption and fluorescence emission spectra in the THF/H_2_O solvent mixture with different water fractions (*f*_w_ = 0 to 99%) (Fig. 2b–d). The UV–Vis absorption spectra of TPE-kana 1 in THF and THF/H_2_O are depicted in Fig. 2b. In THF, TPE-kana **1** exhibits the absorption maxima at around 310 nm wavelength, whereas upon the addition of 99% water, the absorption maxima slightly shifted towards the red. Upon excitation at λ_ex_ = 310 nm, TPE-kana **1** exhibited almost negligible emission in THF solution (Fig. 2c), whereas a sudden increase in the emission was observed at 99/1% of water/THF concentration at 472 nm, due to the formation of aggregates of TPE-kana **1** in water. Hence this significant enhancement in the fluorescence intensity at 99% of water fraction in THF solution of TPE-kana **1**, (Fig. 2c) indicating a strong AIE effect and the quantum yield also increases from 0.3 to 25% for 0/100 to 99/1% of water/THF concentration. The change in emission intensity with % of water in THF is illustrated in Fig. 2d. These results indicated that TPE-kana **1** possessed excellent AIE characteristics and exhibited the highest fluorescence intensity in THF/H_2_O (*f*_w_ = 99%), the SEM image is shown in ESI Fig. S13.

## Sensing performance of TPE-kana 1

**Figure 3:**
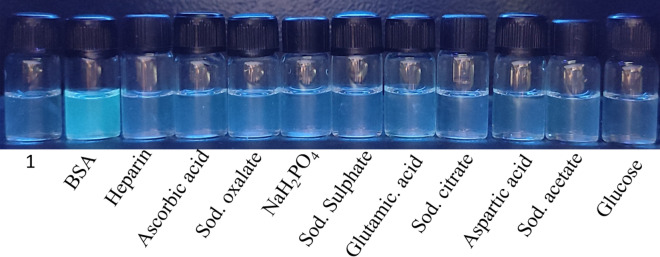
Picture of TPE-kana **1** in in distilled water with and without addition of BSA and other biological competitors under UV light 365 nm.

The stock solution of the TPE-kana **1** (5 × 10^−5^ mol/L) was prepared by dissolving in DMSO solvent. The stock solution from DMSO was used to prepare solution of TPE-kana **1** in distilled water. A series of biomolecules (4 equiv.) such as BSA, heparin, ascorbic acid, sodium pyrophosphate, sodium oxalate, sodium citrate, adenosine triphosphate, glutamic acid, aspartic acid, sodium acetate, and glucose were added to TPE-kana **1** in water. The sensing performance of TPE-kana **1** was then monitored by the color change in the UV light (365 nm) and is depicted in Fig. 3. Under UV light the blue fluorescence of the blank changes to slightly green in the presence of BSA, whereas TPE-kana **1** did not show such color change in presence of other biomolecules, as shown in Fig. 3. These naked eye results suggested that TPE-kana **1** as a fluorescent sensor can be used for selective detection of BSA in solution.

Furthermore, we have examined sensing capability of TPE-kana **1** in distilled water using UV–Vis absorption, fluorescence spectroscopy and also studied competitive experiments to figure out the selectivity of TPE-kana **1** toward BSA. The theoretical docking studies employed to investigate interaction of TPE-kana **1** with BSA either site I and/or site II or within them.

### UV–Vis absorption study

**Figure 4:**
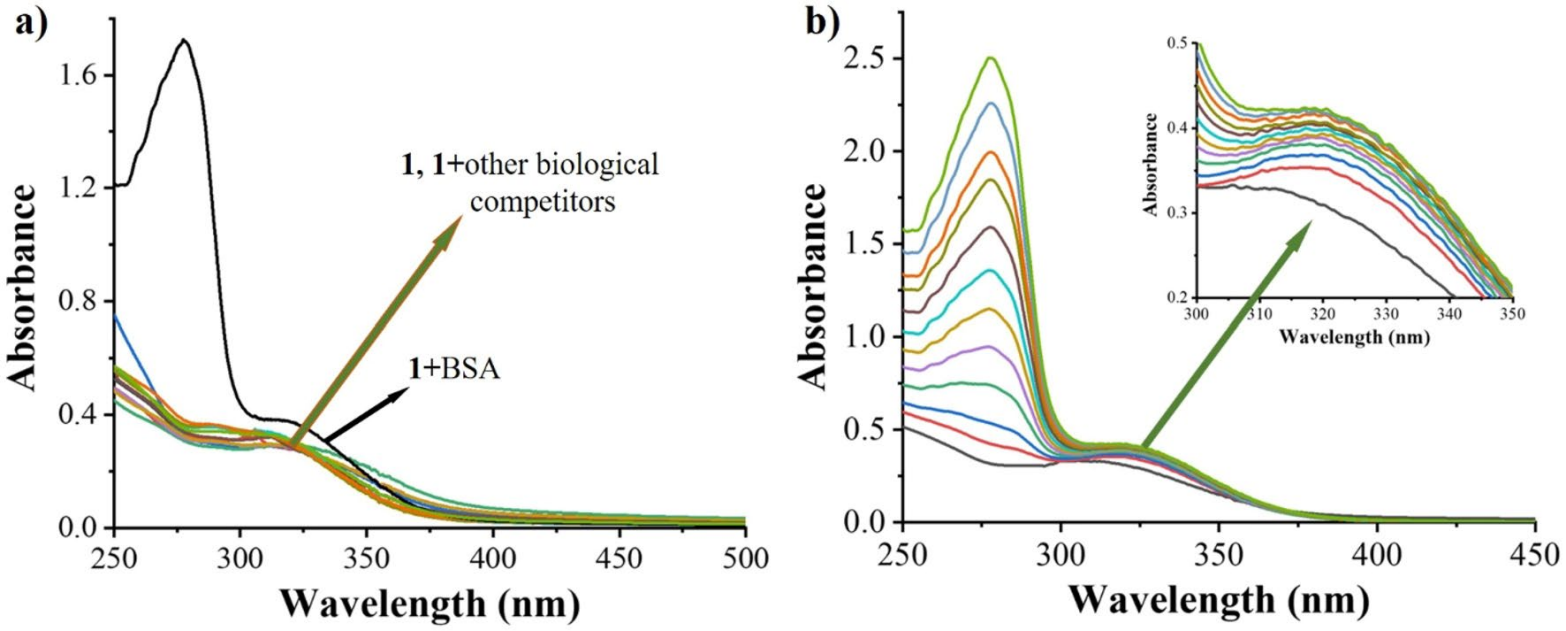
UV–vis absorption spectra of probe TPE-kana **1** (5 × 10^−5^ M) in distilled water upon addition of (**a**) 120 nM of BSA, heparin, ascorbic acid, sodium pyro-phosphate, sodium oxalate, sodium citrate, adenosine triphosphate, glutamic acid, aspartic acid, sodium acetate and glucose. (**b**) Addition of the BSA (0–120 nM) in D/W.

The UV–Vis absorption spectroscopy was employed to investigate the sensing response of TPE-kana **1** towards biomolecules. TPE-kana **1** shows selectivity towards BSA protein only as compared with other biomolecules/proteins as shown in Fig. 4a. Typically, in aqueous solution TPE-kana **1** (5 × 10^−5^ M) exhibit absorption maxima centred at 306 nm, and which upon BSA addition a new absorption band appeared near 280 nm wavelength with the enhanced intensity and broaden peak at 306 nm and no such effect occurred with various other biological competitors as shown in Fig. 4a. These results clearly demonstrated that the non-covalent interactions between TPE-kana **1** with BSA not only through intermolecular hydrogen-bonding between the amino- and hydroxyl-functional group of TPE-kana **1** with BSA but also packing of the TPE-kana **1** in the pocket of BSA, which leads to change in the optical properties of TPE-kana **1**. For a detailed understanding, titration of TPE-kana **1** with BSA (0–120 nm) in distilled water (D/W) was evaluated, which was monitored at absorbance band i.e., 280 nm and 306 nm band as shown in Fig. 4b. Which clearly shows increasing the absorbance band at 280 nm along with broadening band at maxima at 306 nm. Thus, UV–Vis absorption changes clearly shows the TPE-kana **1** shown to be excellent candidate towards selective sensing of BSA.

### Fluorescence emission study

**Figure 5:**
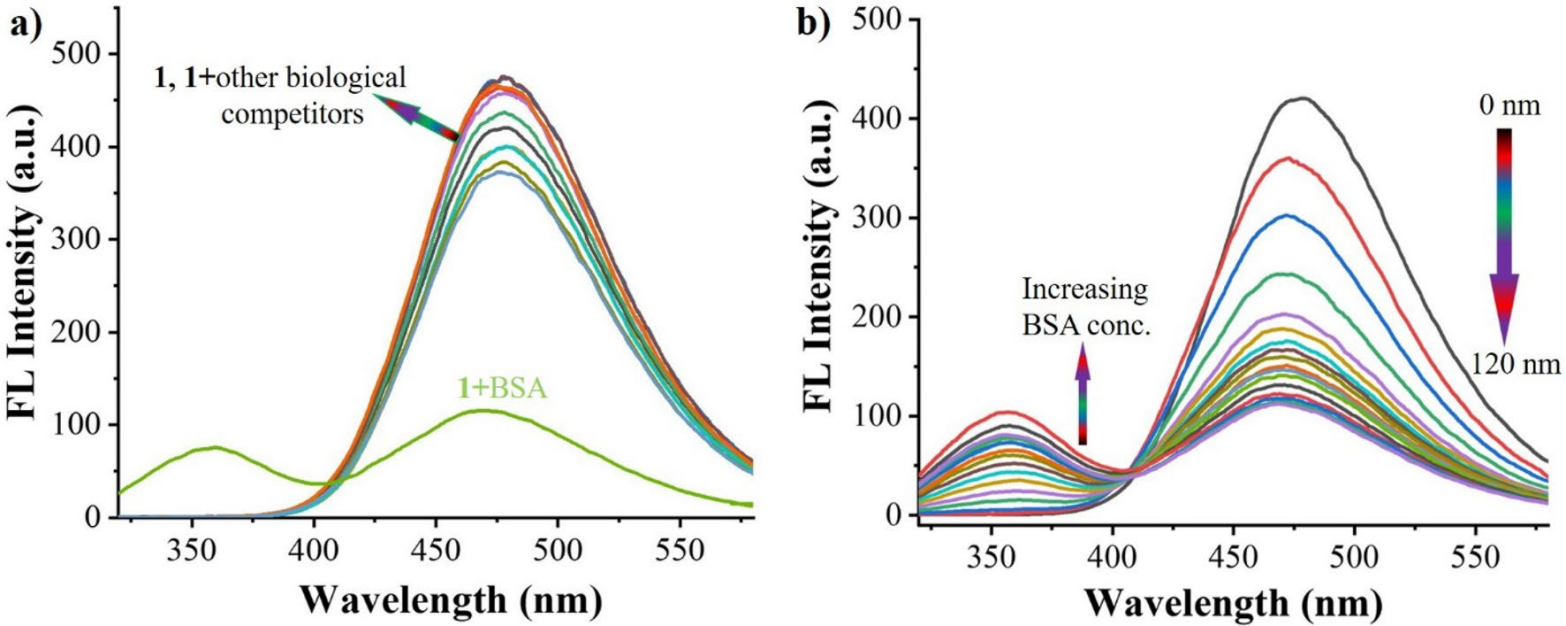
Emission spectra of probe **1** (5 × 10^−5^ M) in water with the addition of (**a**) 120 nM of BSA, heparin, ascorbic acid, sodium pyro-phosphate, sodium oxalate, sodium citrate, adenosine triphosphate, glutamic acid, aspartic acid, sodium acetate and glucose (**b**) addition of BSA (0–120 nM).

A fluorescence spectroscopy was used to investigate the effect of BSA on changes occurred in AIE-active TPE-kana **1** (stock solution was prepared in DMSO) through detection in distilled water at room temperature. The fluorescence spectra of TPE-kana **1** (5 × 10^−5^ M) exhibited typical emission band at 476 nm upon excitation at λ_ex_ = 310 nm as shown in Fig. 5a. The quantum yield (Φ) of the TPE-kana **1** estimated 0.42 in D/W at room temperature. The selectivity TPE-kana **1** towards BSA with the addition of a series of anions and biomolecules was monitored in the changes at 476 nm emission band. It clearly shows no significant changes with the addition other competitors except BSA, in particularly upon addition of BSA the emission intensity of TPE-kana **1** at 476 nm dramatically decreased with appearance of a new emission band at 357 nm. Further, upon gradual incremental addition of BSA (0–120 nM) over the solution of TPE-kana **1** in D/W, the fluorescence emission gradual decrease 476 nm and enhanced emission at 375 nm, Fig. 5b. The quantum yield of the TPE-kana **1**: BSA was found to be decreased to 0.17 as compared with TPE-kana **1** (Φ = 0.47). The emission intensity at 476 nm considerably decreased from 422 to 107 as the concentration of BSA increased from 0 to 120 nM and further additional of BSA does not show any more changes may be due to the detection limit. This clearly suggest the interaction of TPE-kana **1** with BSA protein over other biological competitors almost silent, because of hydrophobic interactions along with H-bonding between –OH and –NH_2_ group of kana moiety and amino acids of BSA followed by π–π interactions between tryptophan amino acid of BSA and TPE unit of TPE-kana **1**. We believe that the strong H-bonding between kanamycin of TPE-kana **1** bind within the molecular cleft of BSA, which can be clearly seen in changes under UV–Vis absorption (Fig. 4) and fluorescence emission spectroscopy (Fig. 5) and later docking study confirm interactions.

The initial fluorescence intensity of TPE-kana **1** was found to show a significant decrease upon incremental addition of the BSA. Therefore, fluorescence quenching efficiency (η) was calculated using equation [(I_0_ − I)/I_0_] × 100%, where I_0_ and I are the fluorescence intensities before and after addition of the BSA. After the addition of 120 nM of BSA solution the initial emission intensity of TPE-kana **1** was quenched by approximately 71.16%, the quenching efficiency (η) was calculated to be about 71.16%.

### Selectivity study of 1 for BSA

**Figure 6:**
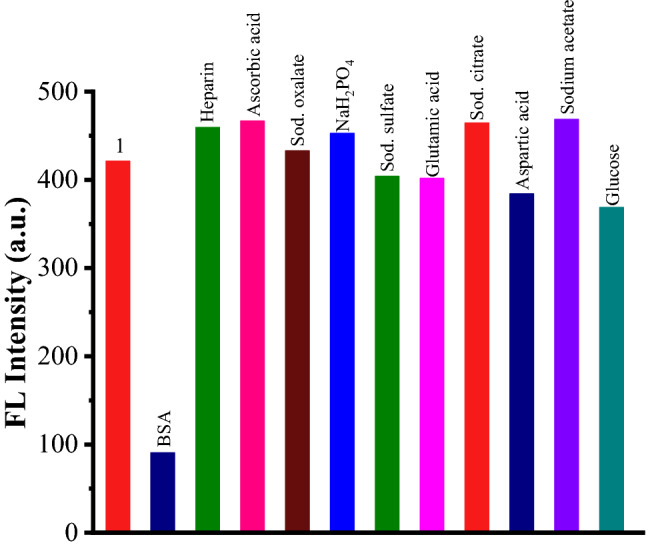
Fluorescence emission responses of TPE-kana **1** toward BSA (120 nM) and its other biological competitors (120 nM) at 476 nm in D/W.

To evaluate the selectivity study of TPE-kana **1** for BSA detection, the fluorescence response of TPE-kana **1** with several anions (120 nM) as well as biological competitors (120 nM) of BSA such as heparin, ascorbic acid, sodium pyrophosphate, sodium oxalate, sodium citrate, adenosine triphosphate, glutamic acid, aspartic acid, sodium acetate, glucose was investigated. TPE-kana **1** did not show a considerable response neither to the anions and not to biological competitors to BSA as illustrated in Fig. 6. It clearly shows selective detection of BSA over other biological competitors.

### Stern–Volmer quenching constant and limit of detection

**Figure 7:**
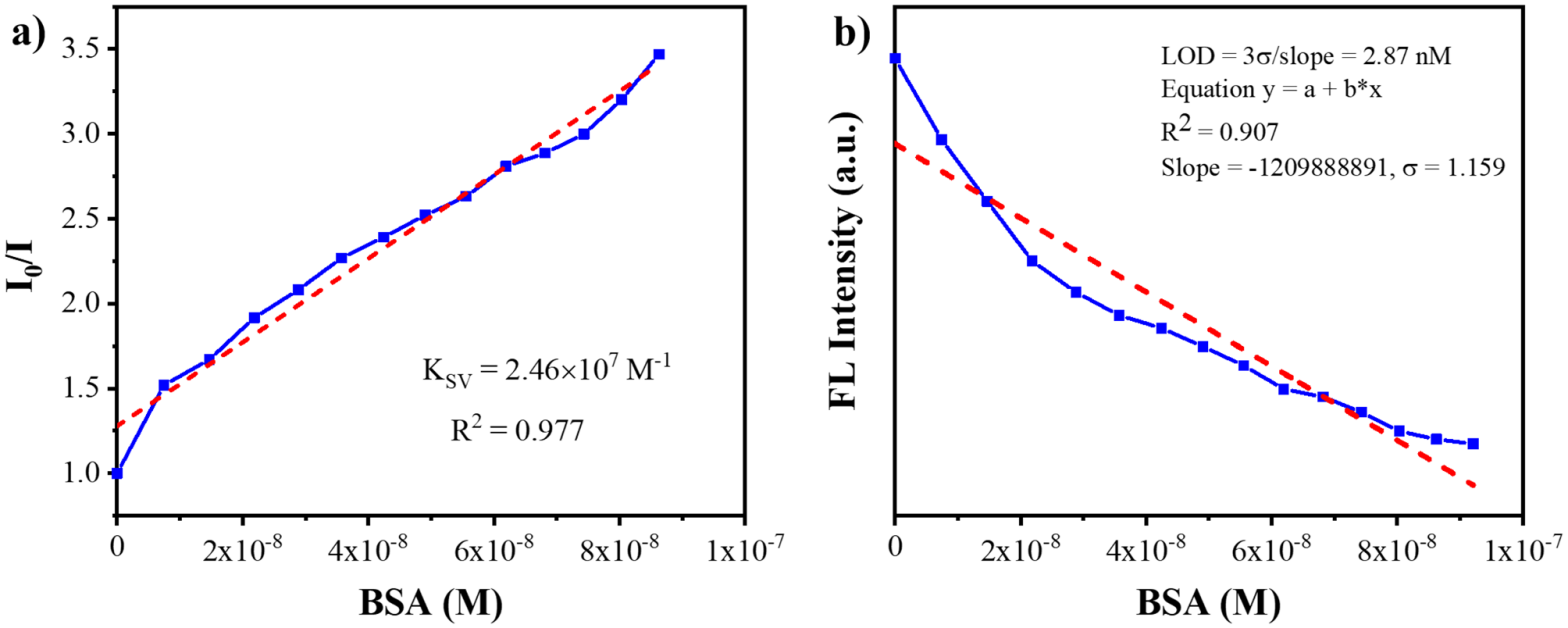
(**a**) Stern–Volmer plot for TPE-kana **1** with BSA and (b) Plot for the determination of the limit of detection (LOD) for BSA.

The Stern–Volmer quenching constant (K_sv_) was calculated by employing fluorescence emission intensity (I_0_/I) as a function of increasing BSA concentration [Q] by the following relation; I_0_/I = TPE-kana **1** + K_sv_ [Q], where I_0_ and I are the emission intensities of TPE-kana **1**, before and after addition of BSA, respectively, K_sv_ is the quenching constant (M^−1^), and [Q] is the molar concentration of BSA. The Stern–Volmer plot of TPE-kana **1** with BSA is showed in Fig. 7a, it can be seen that Stern–Volmer plot followed a good linearity at low concentrations of BSA whereas, at higher concentrations, the linearity slightly deviated as an upwardly bent curve. The K_sv_ value obtained for BSA was 2.46 × 10^7^ M^−1^. This indicated that the BSA exhibited exclusive quenching ability towards fluorescent TPE-kana **1** in water.

To evaluate the detection limit, emission titration of TPE-kana **1** with BSA in distilled water was carried out by adding increasing concentrations of BSA solution (0–120 nM) and the emission intensity as a function of BSA added was then plotted, Fig. 7b. The detection limit was calculated by using the equation as follows:

Detection limit = 3 *σ*/*m.*

Where σ is the standard deviation of the emission of the free sensor and *m* is the slope between emission at 476 nm and concentration of BSA. The detection limit for BSA was shown to be 2.87 nM (R^2^ = 0.907) in distilled water showing the fluorescence intensity of TPE-kana **1** at 476 nm (λ_ex_ = 310 nm) as a function of BSA concentration. Hence the detection limit of TPE-kana **1** in distilled water was found to be 2.87 nM which is higher or comparable with other molecules used for detection of BSA (Table S1).

### Binding constant

Furthermore, the Benesi–Hildebrand plot (ESI Fig. S14), was used to define the binding constant (K_a_) between **1** and BSA. The linear relationship of absorption intensity as a function of 1/[BSA] was found to be 7.56 × 10^7^ M with R^2^ = 0.9968. This suggest that the TPE-kana **1** to have strong binding affinity towards BSA. Furthermore, SEM images clearly shows strong interaction between TPE-kana** 1** with BSA, which produces highly packed coagulated microstructure (Fig. S15) as compared with TPE-kana 1 only produces particular aggregates (Fig. S13) in in distilled water.

## Molecular docking study

**Figure 8:**
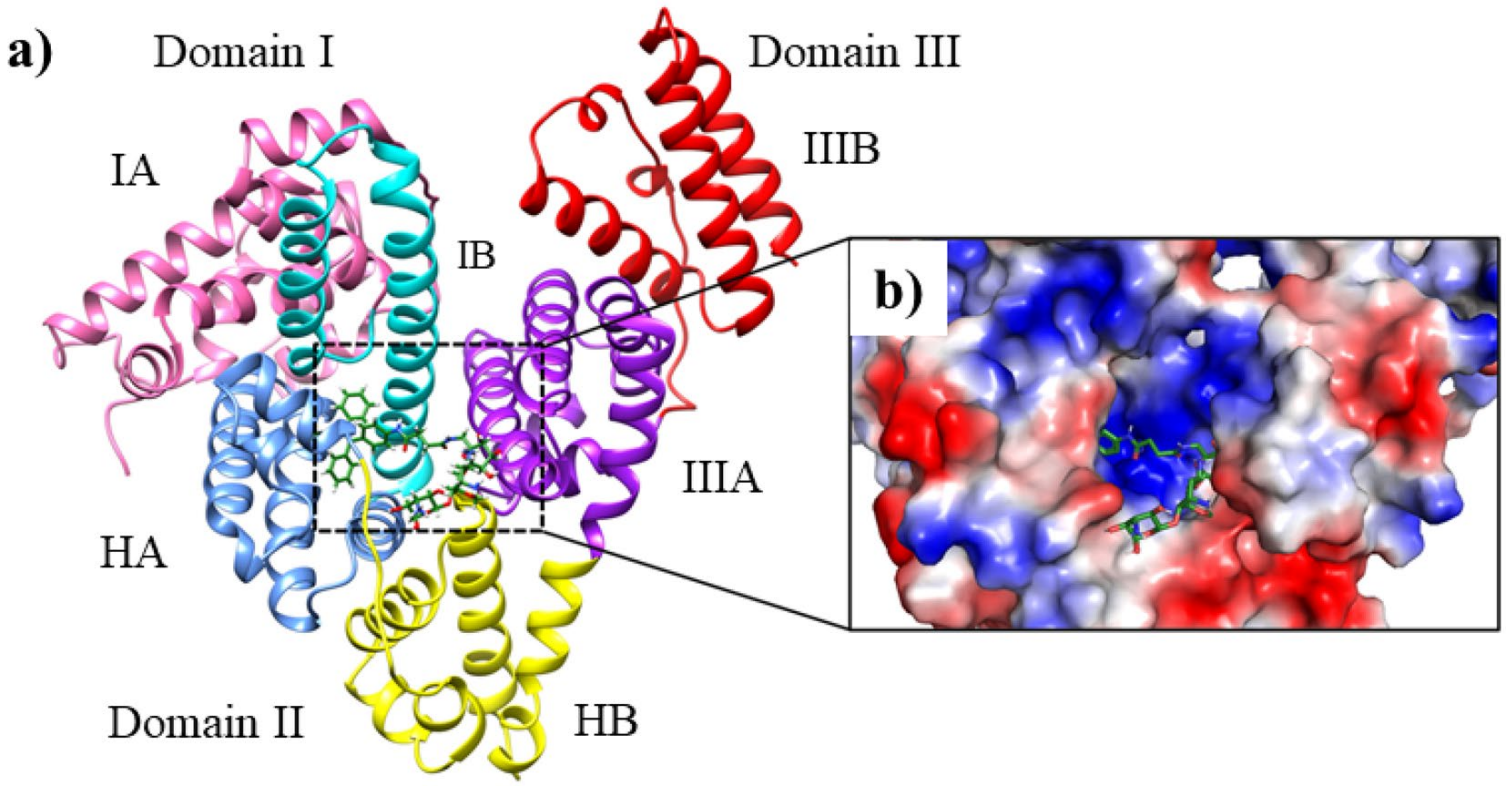
Show the docked complex of BSA with TPE-kana **1**. Here, (**a**) shows the least energy docked conformation of TPE-kana **1** between the domains I and II. The TPE-kana **1** is shown in the stick model and carbon, hydrogen, nitrogen, and oxygen in green, grey, blue and red color, respectively. (**b**) Shows zoomed view of TPE-kana **1** binding pocket of BSA protein with electrostatic surface area. The images (**a**) and (**b**) were generated using the Chimera (pettersen 2004) and PyMol software, respectively^33,34^.

**Figure 9:**
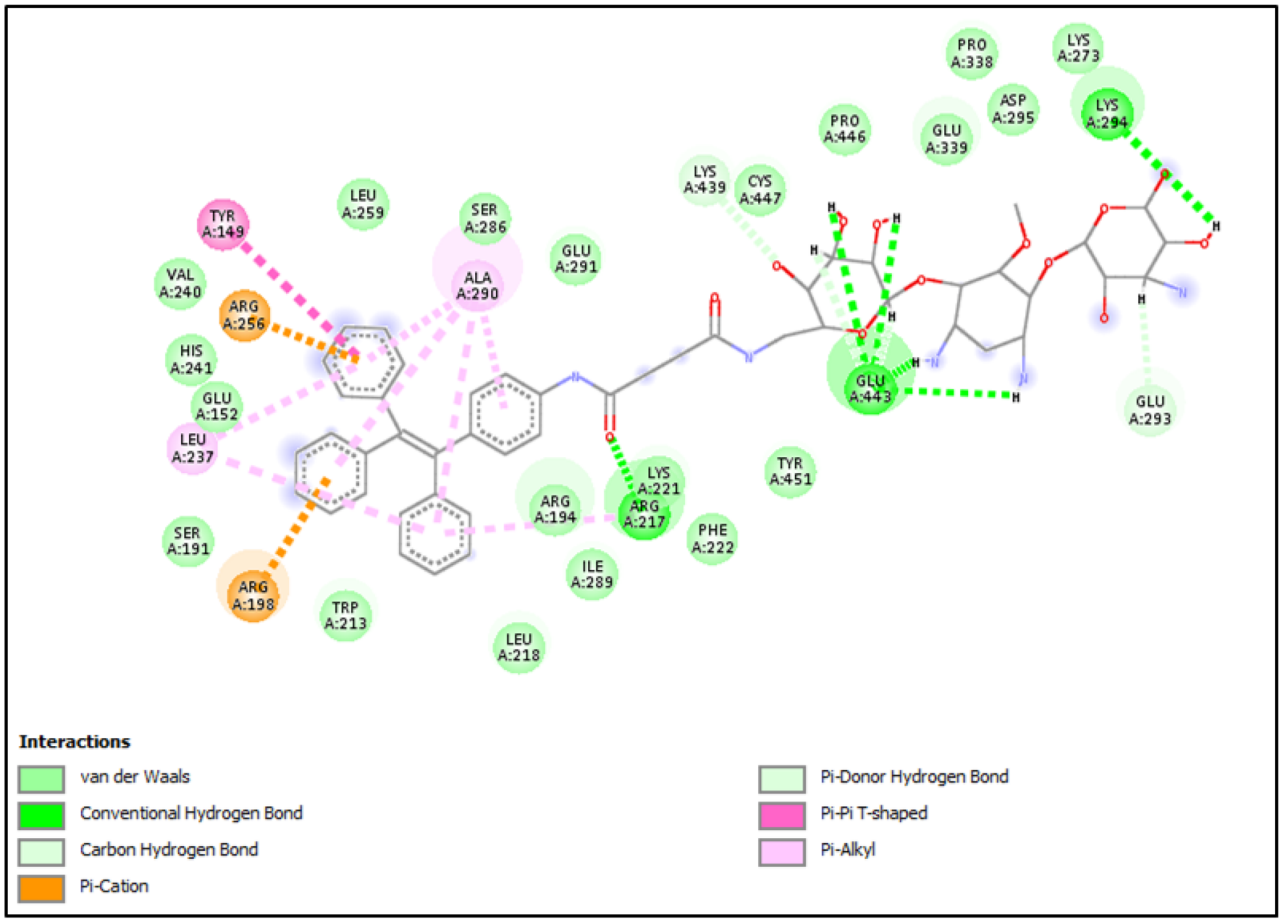
Shows the 2D interactions network of BSA residues with TPE-kana **1**. Here, TPE-kana **1** forms conventional hydrogen bonding, carbon-hydrogen, van der Waals, and π-type of interactions with the binding pocket residue of BSA protein. The 2D interaction network image was generated using Discovery studio Visualizer (BIOVIA, 2016)^35^.

To evaluate binding mode of TPE-kana **1** with BSA, molecular docking study using AutoDock4.2.6^32^ was employed. The TPE-kana **1** shows significant binding affinity with the BSA protein and the lowest binding energy conformation was found at − 10.42 kcal/mol and shown in Fig. 8. The binding site of TPE-kana **1** at the sub-domains IIA of site 1 was analyzed with the least binding energy conformation of TPE-kana **1**. To understand the bonding and non-bonding interactions of TPE-kana **1** with BSA, we further analyzed the docked complex. The analysis of BSA with TPE-kana **1** complex shows the conventional hydrogen bonding interactions with BSA residues such as Glu443 (2.01 Å, 1.84 Å, 1.73 Å, 2.96 Å), Lys294 (2.21 Å), Arg217 (1.67 Å) as shown in Fig. 8a. Lys439, Glu293 forms non-conventional hydrogen bonding interaction with TPE-kana **1** as shown in Fig. 8a. In addition, Arg198, and Arg256 forms π-Cation, Tyr149 forms π-π type of interaction, Leu237, and Ala290 forms π-type of interactions, Trp-213 forms π-donor hydrogen bond as shown in Fig. 8a. Whereas, Glu152, Ser191, Arg194, Leu218, Phe222, Val240, His241, Leu259, Ser286, Ile289, Glu291, and Tyr451as shown in Fig. 8b. As shown in Fig. 9, TPE-kana **1** forms conventional hydrogen bonding, carbon-hydrogen, van der Waals, and π-type of interactions play the major role for the binding pocket residue of BSA protein.

In summary, we have successfully designed and synthesized TPE-functionalized kanamycin antibiotic TPE-kana **1** as a molecular probe for selective and sensitive detection of BSA. TPE-kana **1** showed significant AIE properties in THF:water solvent systems. Furthermore, TPE-kana **1** exhibited BSA induced decreased fluorescence emission intensity in distilled water compared to other examined anions and biological competitors in aqueous medium. The sensing of BSA was well characterized by using UV–Vis absorption and fluorescence emission spectroscopy, the quenching of fluorescence observed with BSA. Moreover, the quenching efficiency was calculated and obtained was 71.16%. The Stern Volmer quenching constant was calculated and found to be 2.46 × 10^7^ M^−1^. The limit of detection of TPE-kana **1** towards BSA was found to be 2.87 nM. The binding constant was calculated and found to be 7.56 × 10^7^ M and R^2^ = 0.9968. The molecular docking study revealed the significant binding affinity of TPE-kana **1** for BSA with the lowest binding energy conformation was found at − 10.42 kcal/mol. These results clearly indicated that TPE-kana **1** may use as a promising recognition tool for the BSA in solution as well as biomedical waste.

## Experimental section

### Materials and methods

Compounds **3** and **4** were synthesized using reported literature procedure and compound **5** and TPE-kana **1** were synthesized using following procedure (Fig. S1). All reagents including toluene, DMSO, acetic acid, hydrazine hydrate, O-(Benzotriazol-1-yl)-N,N,N′,N′-bis(tetramethylene)uronium hexafluorophosphate (HBPyU), Tetrahydrofuran (THF), and kanamycin sulfate and various biomolecules such as BSA, heparin, ascorbic acid, sodium pyrophosphate, sodium oxalate, sodium citrate, adenosine triphosphate, Glutamic acid, aspartic acid, sodium acetate, and glucose were purchased from Sigma-Aldrich, and TCI chemicals. All the reactions were monitored by thin-layer chromatography (TLC) and ^1^H and ^13^C NMR spectra were recorded on 400 MHz and 100 MHz Bruker spectrometer with tetramethylsilane (TMS) as an internal standard and CDCl3-d and MeOD-d4 as deuterated solvents. Thermofisher exactive orbitrap MALDI-TOF measurements were used for HRMS, a Schimadzu Biotech Axima performance spectroscopic instrument. UV–Vis absorption spectra were recorded using a UV–Vis 1800 Shimadzu spectrophotometer and fluorescence emission was measured on an Agilent, Carry Eclipse spectrofluorophotometer.

### General procedure for UV–Vis and fluorescence measurements in aqueous solutions

A stock solution (1 mM) of TPE-kana **1** was prepared in DMSO and was stored in a cold and dark place. The stock solution was used for the experiments after appropriate dilution in distilled water (D/W). The concentration of TPE-kana **1** was confirmed by absorbance at 306 nm for the TPE fluorophore UV–Vis absorption spectra (250–550 nm) of the samples in a 10 mm path length cuvette was measured using a UV–Vis 1800 Shimadzu spectrophotometer fluorescence emission spectra of TPE-kana **1** in a 10 mm path length cuvette were measured using an Agilent, Carry Eclipse Spectro fluorophotometer with excitation at 310 nm. All absorbance and fluorescence measurements were carried out in 100% aqueous solutions at room temperature.

*UV–Vis absorption and fluorescence emission measurements:* UV–Vis absorption and fluorescence emission of the TPE-kana **1** upon the addition of 4 equiv. of BSA and other biological competitors. The 2 mL TPE-kana **1** solution (5 × 10^−5^ mol/L) in distilled water (D/W) was placed in the quartz cell, and the absorption and emission spectra were recorded with the addition of BSA and its other competitor’s different anions (20 × 10^−3^ M). The absorption and emission spectra were completely recorded at room temperature.

*UV–vis absorption and fluorescence emission titration of the TPE-kana 1 upon the addition of BSA:* The 2 mL TPE-kana **1** solution (5 × 10^−5^ mol/L) in D/W was placed in the quartz cell and the fraction of BSA solution (0–120 nM) was added, and the corresponding absorption and emission spectra were recorded at room temperature.

### Naked-eye detection

The DMSO stock solution of the TPE-kana **1** (5 × 10^−5^ mol/L) was used to prepare solution for necked eye experiments by dissolving it in D/W. Then BSA and its other competitors such as heparin, ascorbic acid, sodium pyrophosphate, sodium oxalate, sodium citrate, adenosine triphosphate, Glutamic acid, aspartic acid, sodium acetate, and glucose were added, the photograph were taken under 365 nm UV light.

### Limit of detection

To determine the detection limit, emission titration of TPE-kana **1** with BSA was carried out by adding increasing concentrations of BSA solution (0–120 nM) and the emission intensity as a function of BSA added was then plotted.

### Molecular docking study

To investigate the binding mode of TPE-kana **1** with BSA, we employed molecular docking calculations using AutoDock4.2.6 software (https://autodock.scripps.edu/)35. The crystal structure of BSA was retrieved from the protein database (source code: 4OR0.pdb). Here, chain A was considered for the molecular docking study. The three-dimensional atomic co-ordinate of TPE-kana **1** was generated using the Discovery Studio Visualizer^35^. For the docking study, a grid box size of 80 × 80 × 80 with a spacing of 0.375 Å was built around the active site of BSA which is present in between the domain IIA and IIIA. Next, we employed a local docking protocol, to explore a binding mode of TPE-kana **1**, similar to an earlier study^34^. Here, we keep BSA as rigid and TPE-kana **1** as a flexible molecule. The output docking conformations were generated by applying the Lamarckian Genetic Algorithm (LGA). These output conformations were further clustered using an all-atom RMSD with a cut-off of 4 Å. The clusters were further analyzed based upon binding, van der Waals, and electrostatic energy, etc. The lowest binding energy conformation of TPE-kana **1** was further analyzed for the bonding interactions using PyMol (DeLano 2002) and Discovery Studio visualizer^35^, respectively.

*High-pressure liquid chromatography (HPLC)* Column: INERTSIL-ODS C18, Solvent: ACN: Water, 9:1, Flow rate: 1 mL/min, Run time: 30 min.

## Supplementary Information

Supplementary Information.

Supplementary Legends.
