# Supplementary material for: Aminoglycoside antibiotic kanamycin functionalized tetraphenylethylene molecular probe for highly selective detection of bovine serum albumin protein
Source: Sci Rep. 2022 Jul 7;12:11526. doi: 10.1038/s41598-022-15890-4 (PMC9263133; doi:10.1038/s41598-022-15890-4)
Supplement: Supplementary file 1 — Supplementary Information. [file 41598_2022_15890_MOESM1_ESM.docx]

**Electronic Supporting Information**

**Aminoglycoside Antibiotic Kanamycin Functionalized Tetraphenylethylene Molecular Probe for Highly Selective Detection of Bovine Serum Albumin Protein**

Ratan W. Jadhav,^1^ Sopan M. Wagalgave,^2,3^ Bajarang V. kumbhar,^4^ Rushikesh M. Khadake,^5^ Ambadas B. Rode,^5^ Sidhanath V. Bhosale,^2,3^ and Sheshanath V. Bhosale^1^*

^1^School of Chemical Sciences, Goa University, Taleigao Plateau, Goa 403206 India. ^2^ Polymers and Functional Materials Division CSIR-Indian Institute of Chemical Technology, Hyderabad 500007, Telangana, India. ^3^Academy of Scientific and Innovative Research (AcSIR), Ghaziabad-201002, India. ^4^Department of Biological Sciences, Sunandan Divatia School of Science, NMIMS (Deemed to be) University, Vile Parle, Mumbai- 400057, India. ^5^Laboratory of Synthetic Biology, Regional Centre for Biotechnology, Faridabad, Haryana-121001, India. *corresponding author E-mail: [svbhosale@unigoa.ac.in](mailto:svbhosale@unigoa.ac.in)

**Synthesis of TPE-kana 1:**

**Figure S1.**Synthetic route for compound TPE-kana **1**.

**Synthesis of 1.** Compounds **3** and **4** were synthesized according to the previously reported procedure. Compound **5** was synthesized by reacting **4** with succinic anhydride in toluene solvent and amide coupling between the **5** and kanamycin sulfate in THF/water mixture (1:1) as a solvent and HBPyU as a coupling reagent resulted in the formation of **1**, white solid, 60% yield. The compound TPE-Kana **1** was characterized by several techniques, such as IR, NMR, HRMS, MALDI-TOF, HPLC and optical rotation. **HRMS:** (m/z) calculated 912.40256 found 912.40365 [M]^+^, 980.28106 [M+3Na]^+^. The purity of TPE-Kana **1** was analyzed by HPLC, above 95% purity was obtained indicating high purity of compound TPE-kana **1**, Column: INERTSIL-ODS C18, Solvent: ACN:Water, 9:1, Flow rate: 1mL/min, Run time: 30 min. The specific rotation for the TPE-kana 1 was calculated using polarimeter, the obtained specific rotation is α^23^_D_= ̶ 1.4929 (c 0.7, DMSO).

**Synthesis of 3:** The compound **3** was synthesized by using the reported literature procedure^S9^: Placed tetraphenylethylene **2** (2.64 g, 8 mmol) then added glacial acetic acid (1.9 mL, 32 mmol), and dichloromethane (80 mL) and reaction mixture is cooled to -15 °C using ice-salt bath after cooling added conc. nitric acid (1.6 mL, 24 mmol) while vigorous stirring. Stirred the reaction mixture for further 15 min maintaining the -15 ^o^C temperature and the progress of the reaction was monitored by TLC. After complition, the reaction mixture was quenched with cold water and separate the organic phase and wash with water three times (3×50 mL). The organic phase was dried over anhydrous Na_2_SO_4_ and filtered. Evaporate the solution to dryness under vacuum to get yellow solid, 2.70 gm, 90% yield. ¹H NMR (CDCl_3_, 400 MHz) δ ppm: 7.95 (2H, d, *J* = 9.1 Hz), 7.14 (11H, m, *J* = 4.0, 11.0, 11.0 Hz), 7.04-6.97 (6H, m).

**Synthesis of 4:** Compound**4** was prepared by literature procedure as follow^S10^:
Firstly 4-nitro-tetraphenylethene (**3**) (1.5 g, 4.0 mmol) was dispersed in EtOH (80 mL) then degassed reaction mixture with nitrogen, thereafter, added 10% Pd/C (0.60 g), and hydrazine-hydrate (6.00 g, 120 mmol). After reflux, the mixture for 4 hours filtered the hot reaction mixture through cellite pad to remove Pd/C. Upon evaporation of reaction mixture under reduced pressure on rotary evaporator gives 4-tetraphenylethenylamine (4) in 1.3 gm (95% yield). ¹H NMR (CDCl_3_, 400 MHz) δ ppm: 7.14-6.98 (15H, m), 6.81 (2H, d, *J* = 8.2 Hz), 6.42 (2H, d, *J* = 8.2 Hz), 3.58 (2H, s).

**Synthesis of 5:** The compound **4** (23 mmol) was treated with succinic anhydride (25 mmol) in dry toluene (20 mL) and refluxed the reaction mixture for 3 h under nitrogen atmosphere. The progress of the reaction was monitored by TLC. After the completion, the reaction mixture cooled to room temperature and filtered the obtained precipitate. White solid, 80% yield. ¹H NMR (CDCl_3_, 400 MHz) δppm: 7.41 (1H, s), 7.24 (2H, d, *J* = 7.7 Hz), 7.12-6.98 (15H, m), 6.96 (2H, d, *J* = 8.0 Hz), 2.77 (2H, t, *J* = 6.2 Hz), 2.64 (2H, t, *J* = 6.5 Hz); FTIR (KBr): ν_max_/cm^-1^ 3309, 3045, 2981, 1919, 1641, 1516, 1435, 1269, 1192, 1068, 835, 721.

**Synthesis of TPE-kanamycin conjugate 1:** Compound 1 was synthesized by modifying reported literature procedure^S11^: Kanamycin sulfate, 130 mg, and NaHCO_3_, 94 mg were dissolved in 10 ml water with stirring. Compound **5**, 100 mg in 10 ml THF was added at room temperature followed by the addition of HBPyU, 97 mg in small portions. The mixture was stirred at room temperature for 6 h. The reaction was quenched with the addition of water, and the precipitate formed was filtered to obtain white solid, 142 mg, 70% yield. ¹H NMR (DMSO-d^6^, 600 MHz) δppm: 7.80 (1H, dd, *J* = 4.8, 9.6 Hz), 7.4 (2H, d, *J* = 8.5 Hz), 7.26 (1H, td, *J* = 2.9, 5.8 Hz), 7.12-7.05 (9H, m), 7.05-7.01 (6H, m), 6.95 (2H, d, *J* = 9.1 Hz), 5.54 (1H, s), 5.14 (2H, d, *J* = 12.5 Hz), 4.10 (4H, m), 3.85 (8H, m), 3.80(2H, m), 3.65(8H, m), 3.55 (6H, m), 3.05 (2H, t, *J* = 6.6 Hz), 2.95 (2H, t, *J* = 7.0 Hz) 1.85 (1H, m); FTIR (KBr): ν_max_/cm^-1^ 3626, 3292, 3062, 2920, 1800, 1660, 1589, 1521, 1408, 1303, 1172, 1062, 763, 696; HRMS (m/z) calculated for C_48_H_60_O_12_N_6_ [M^+^] 912.4025, found 912.4036 (M)^+^; MALDI-TOF (m/z) calculated for C_48_H_60_O_12_N_6_ [M^+^] 912.40, found 936.42 (M+1+Na)^+^, and 1012.52 (M+1+H_2_SO_4_)^+^.


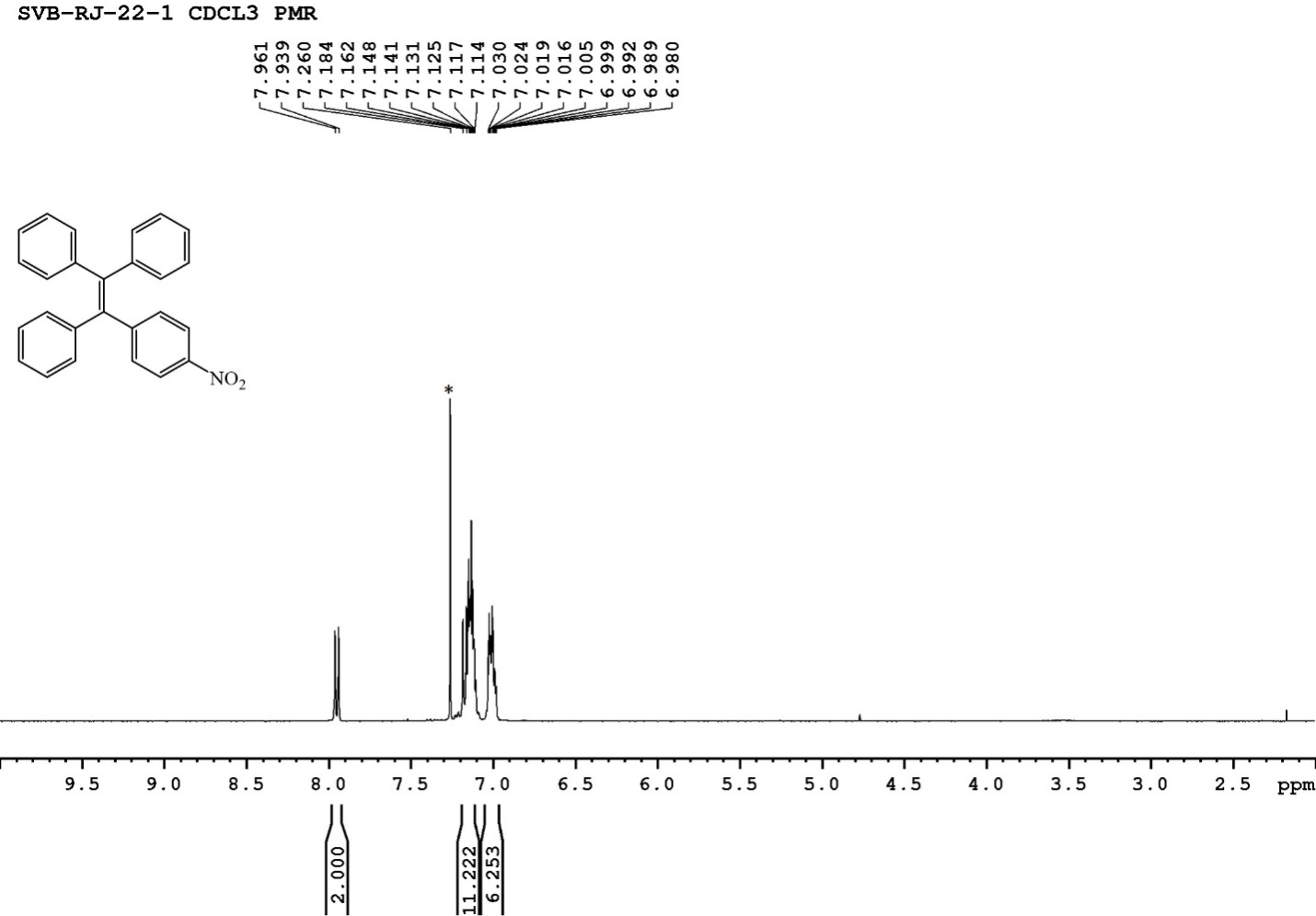
**Figure S2.**^1^H NMR of compound **3** (‘*’ indicates residual solvent peak).


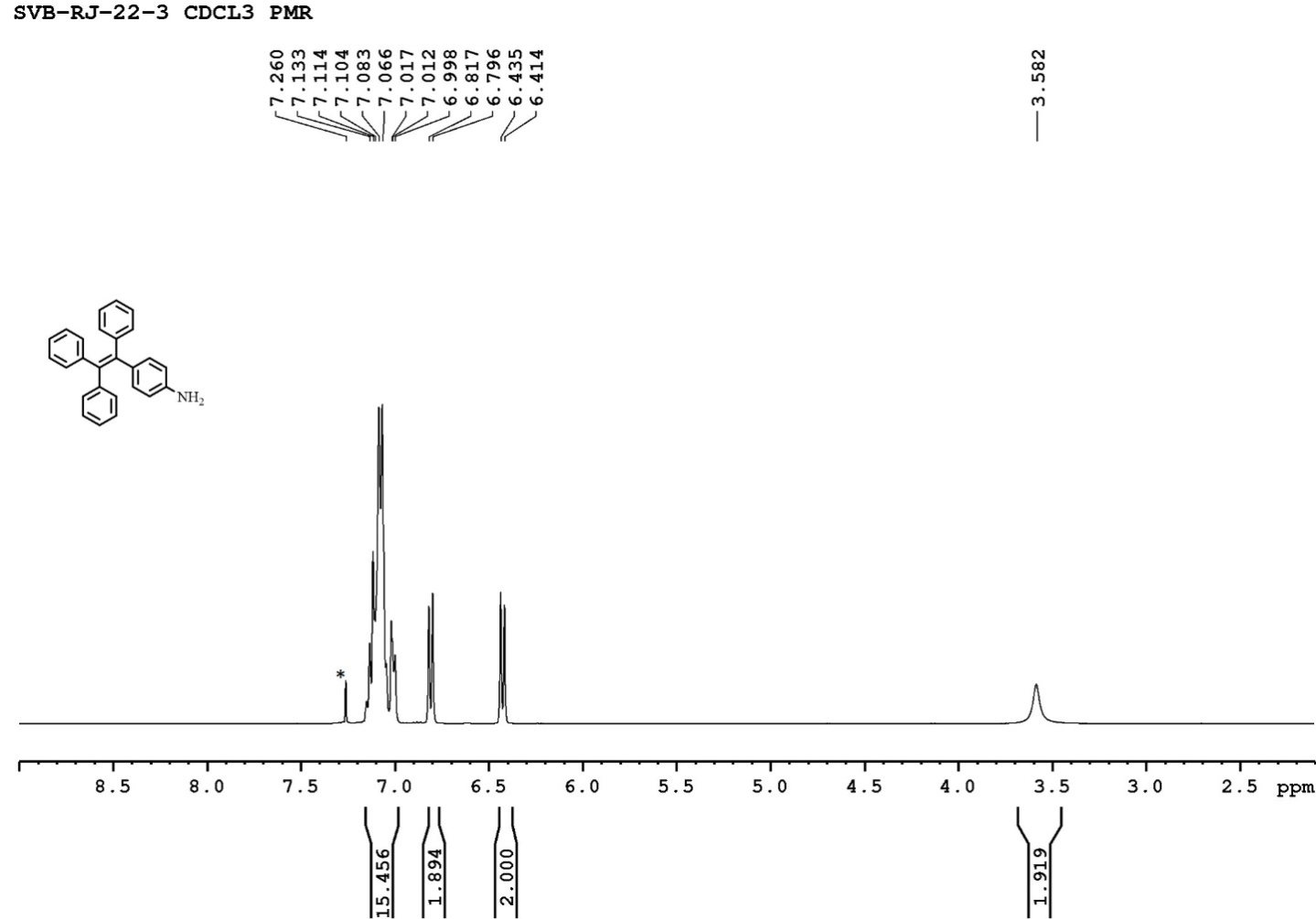


**Figure S3.**^1^H NMR of compound **4** (‘*’ indicates residual solvent peak).

z
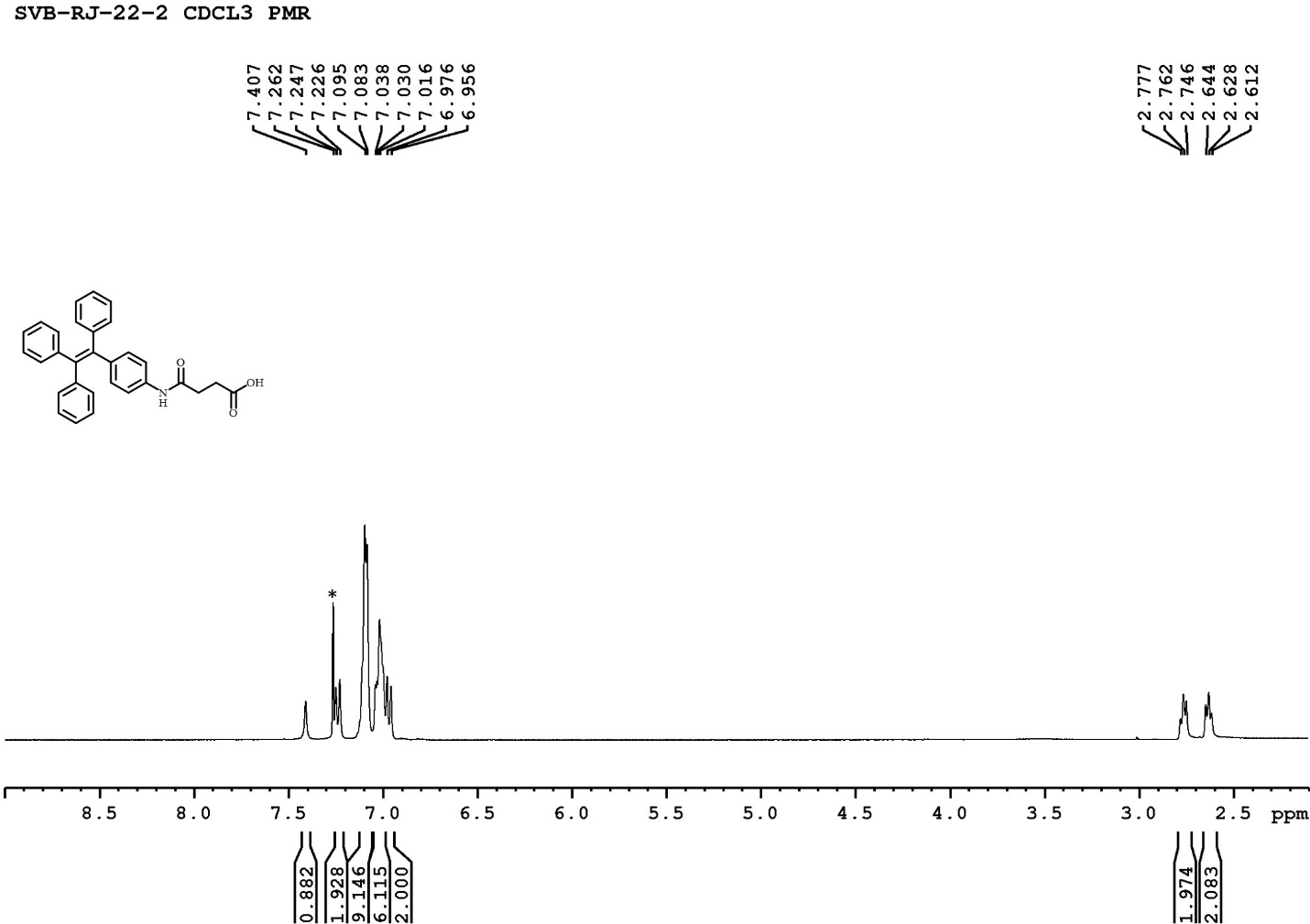


**Figure S4.**^1^H NMR of compound **5** (‘*’ indicates residual solvent peak).


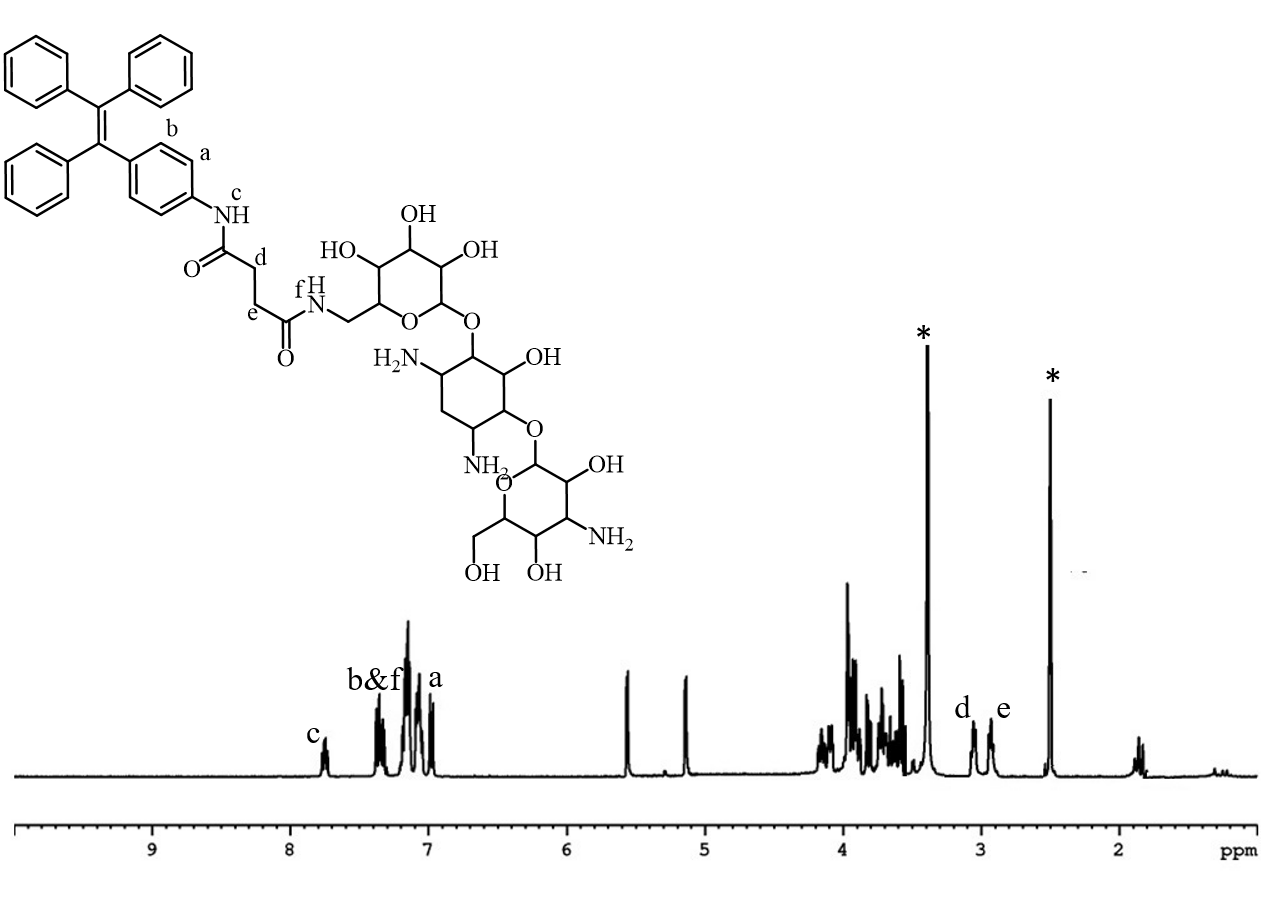
**Figure S5.**^1^H NMR (600MHz, 512 scans) of compound TPE-Kana **1** in DMSO-d_6_.

**
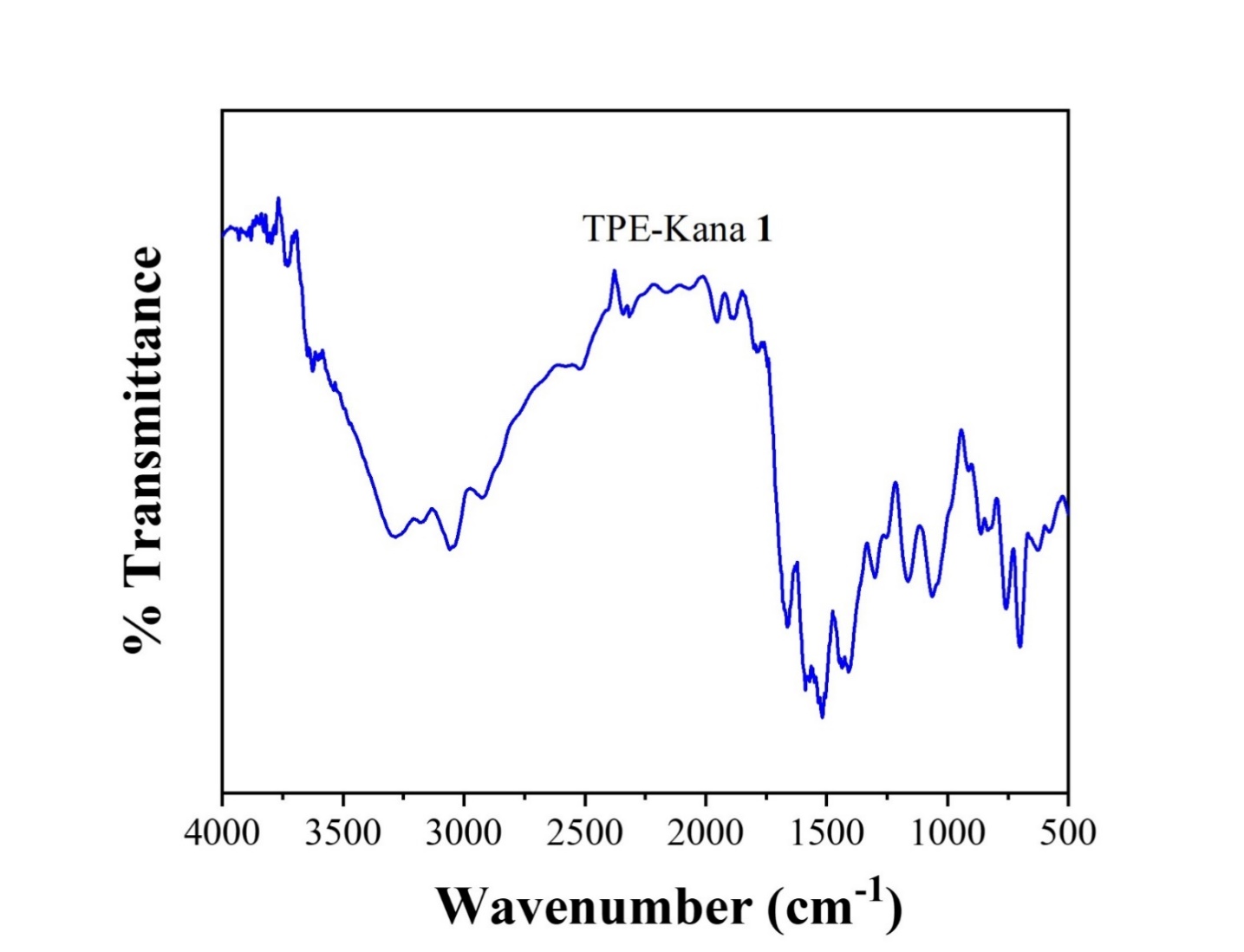
**

**Figure S6.** FTIR of TPE-kana **1.**


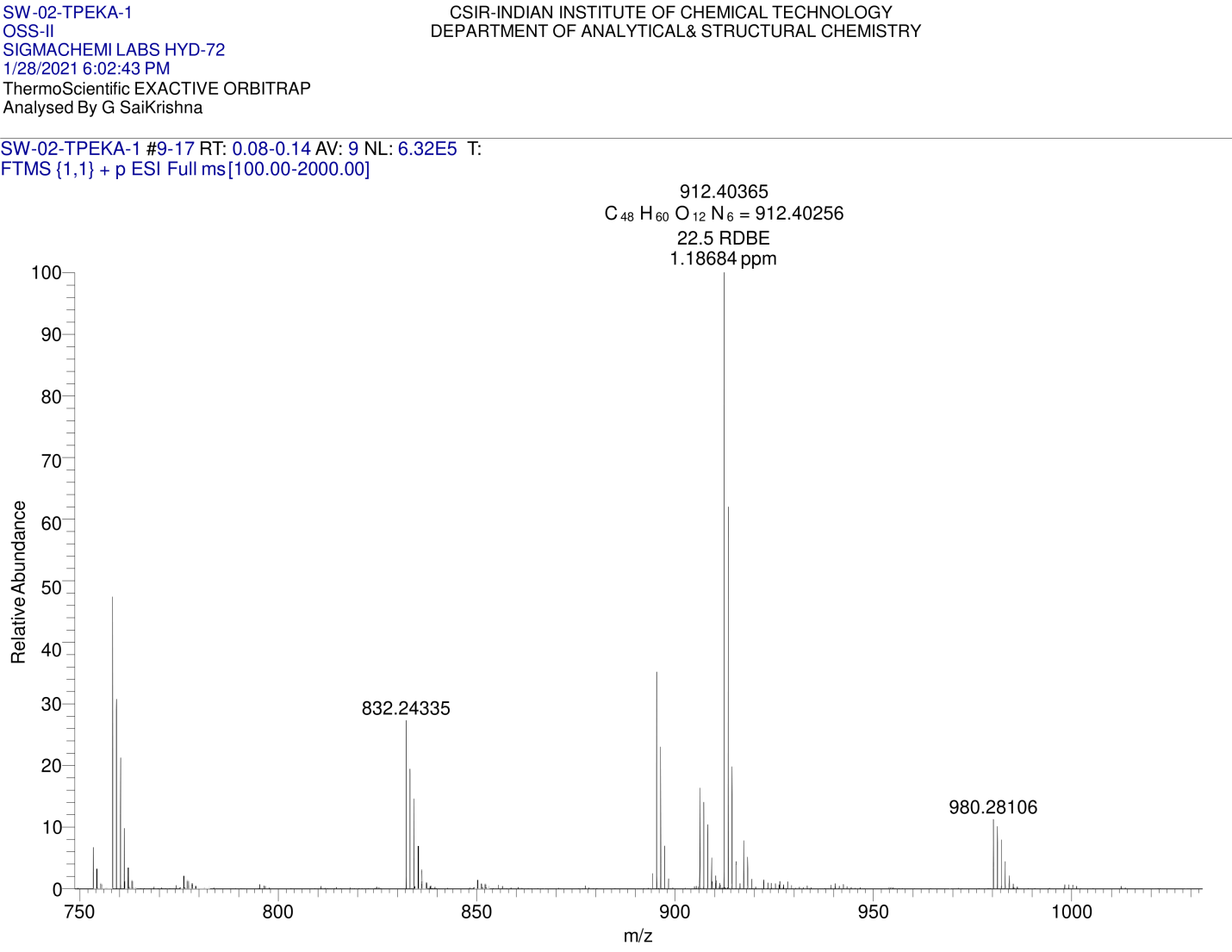
**Figure S7.** HRMS of compound TPE-Kana **1.**


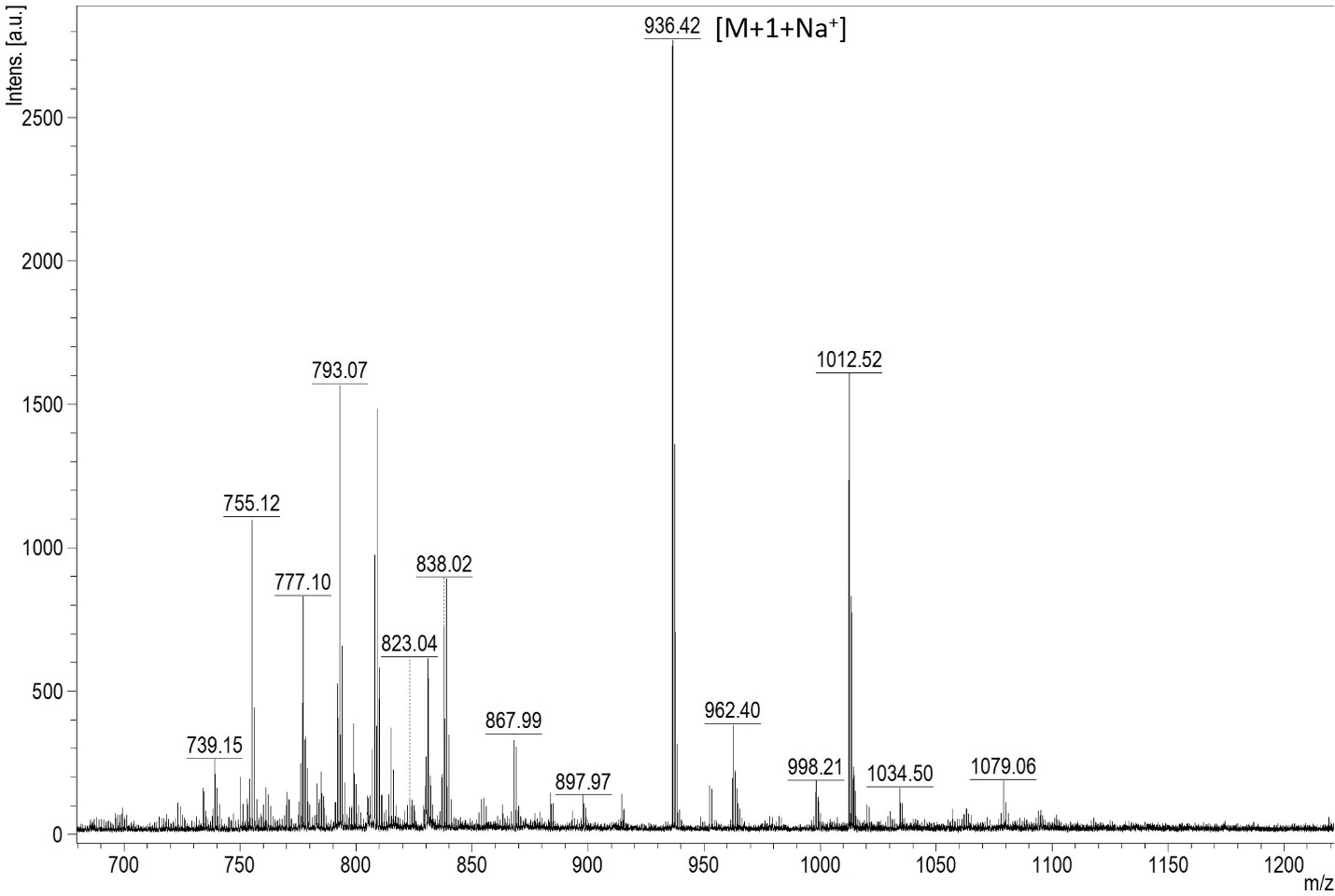


**Figure S8.** MALDI-TOF of compound TPE-Kana **1**

**
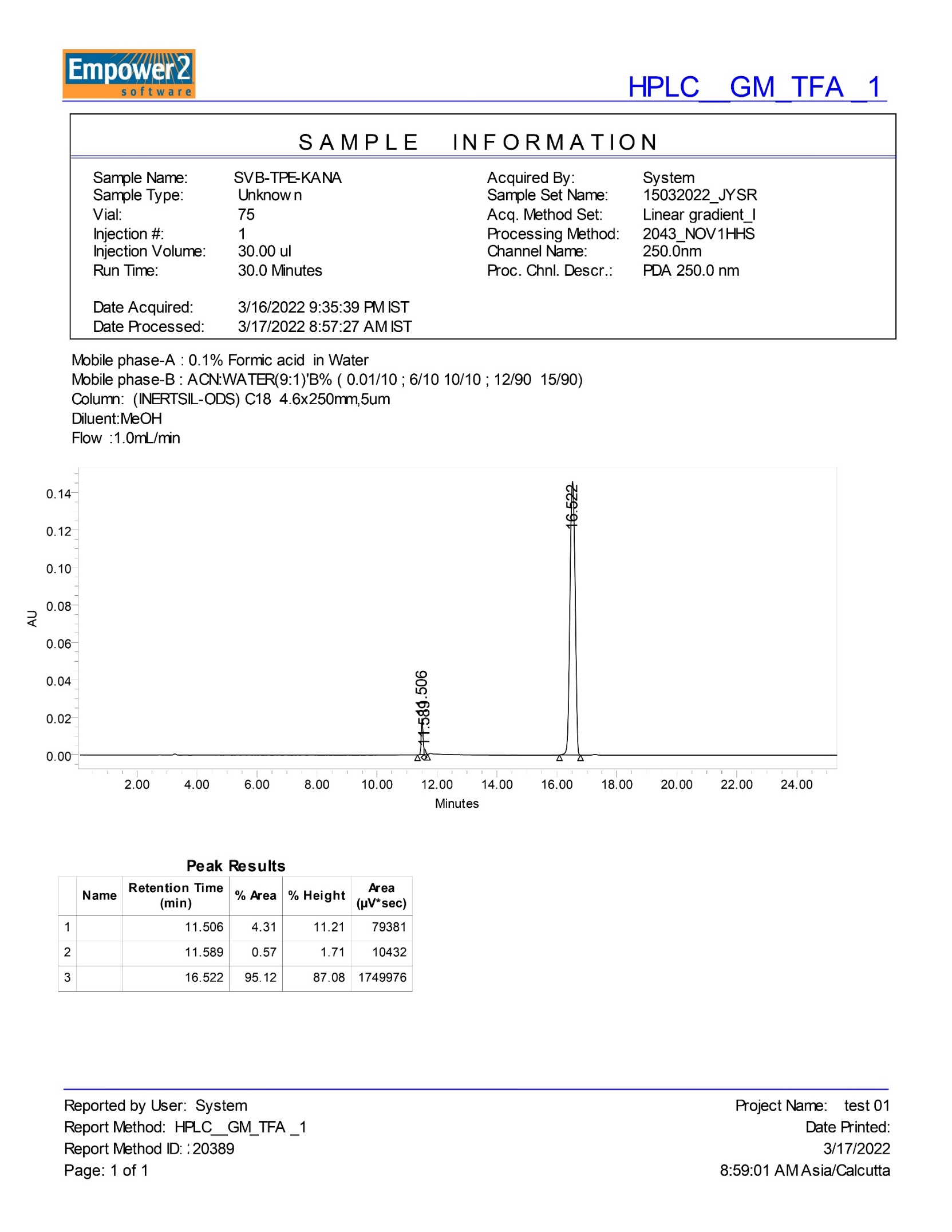
Figure S9.** HPLC of compound TPE-Kana **1.**


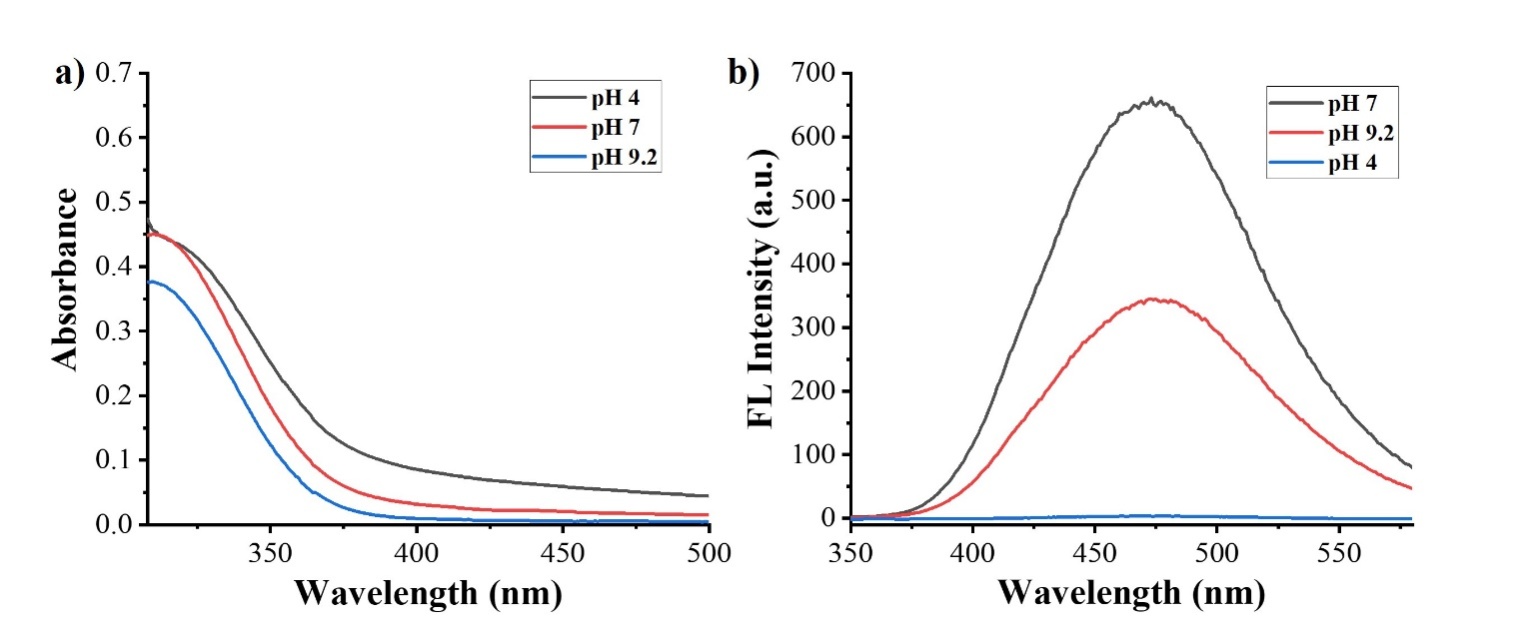


**Figure S10**. a) UV-Vis absorption spectra and b) Fluorescence emission spectra of TPE-kana **1** (10^−5^ M) at different pH (λ_ex_ = 310 nm).


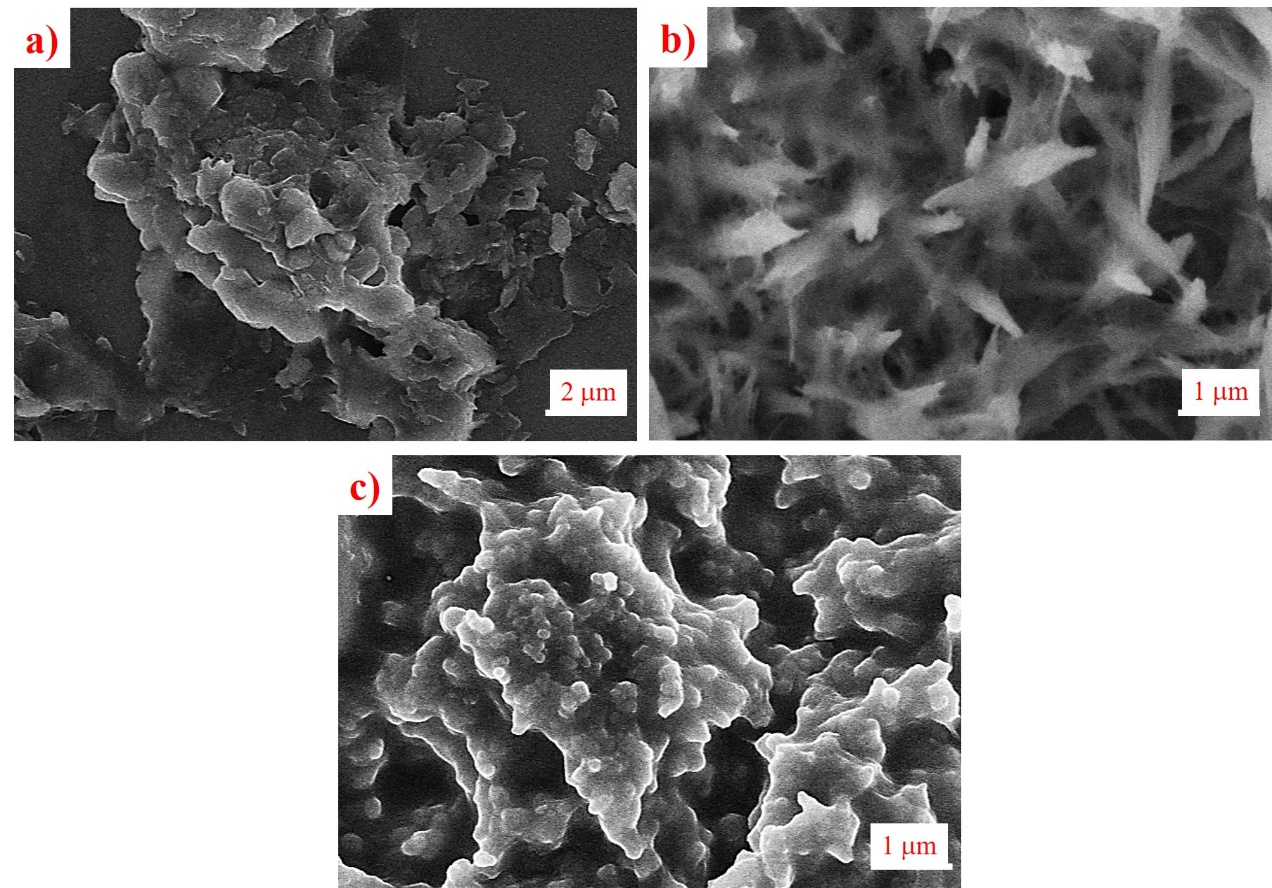


**Figure S11.** SEM images of TPE-kana **1** at a) pH-4, b) pH-7 and c) pH-9.2.


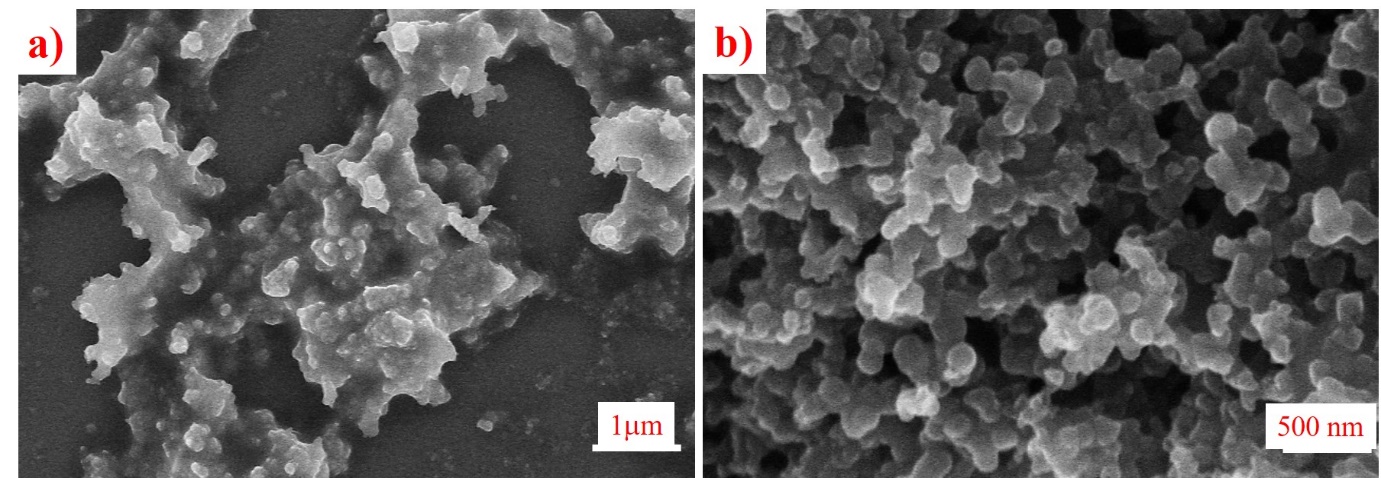


**Figure S12.** SEM images of TPE-kana **1** in THF.


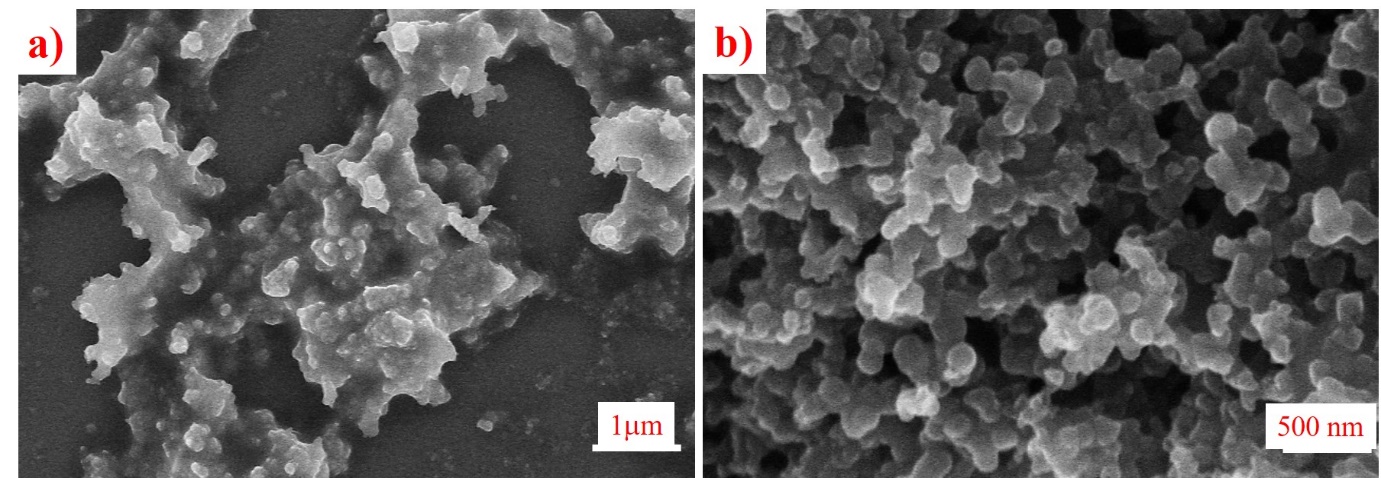


**Figure S13.** SEM images of TPE-kana **1** in 99% water/THF mixture.

**Table S1: comparison of BSA sensing:**

| **Compound** | **Sensing Method** | **LOD** | **Reference** |
| --- | --- | --- | --- |
|  | Fluorescence method | 1 μg/mL | S1 |
|  | Fluorescence method | 0.09 μg/mL | S2 |
|  | Fluorescence method | 5 nM | S3 |
|  | Fluorescence method | 7.3 nM | S4 |
|  | Fluorescence method | 48 nM | S5 |
|  | Fluorescence method | 0.4 μM | S6 |
|  | Fluorescence method | 680nM | S7 |
|  | Fluorescence method | 100 ng/mL | S8 |
|  | Fluorescence & Naked-Eye Detection | 2.87 nM | This  Work |

**Benesi–Hildebrand plot**:

Binding constant of TPE-kana **1** for BSA in aqueous solution was measured based on the linear relationship between the absorption intensity at 305.8 nm and BSA concentration. The binding constant for BSA was measured to be 7.56×10^7^ M (R^2^=0.9968) in an aqueous solution.


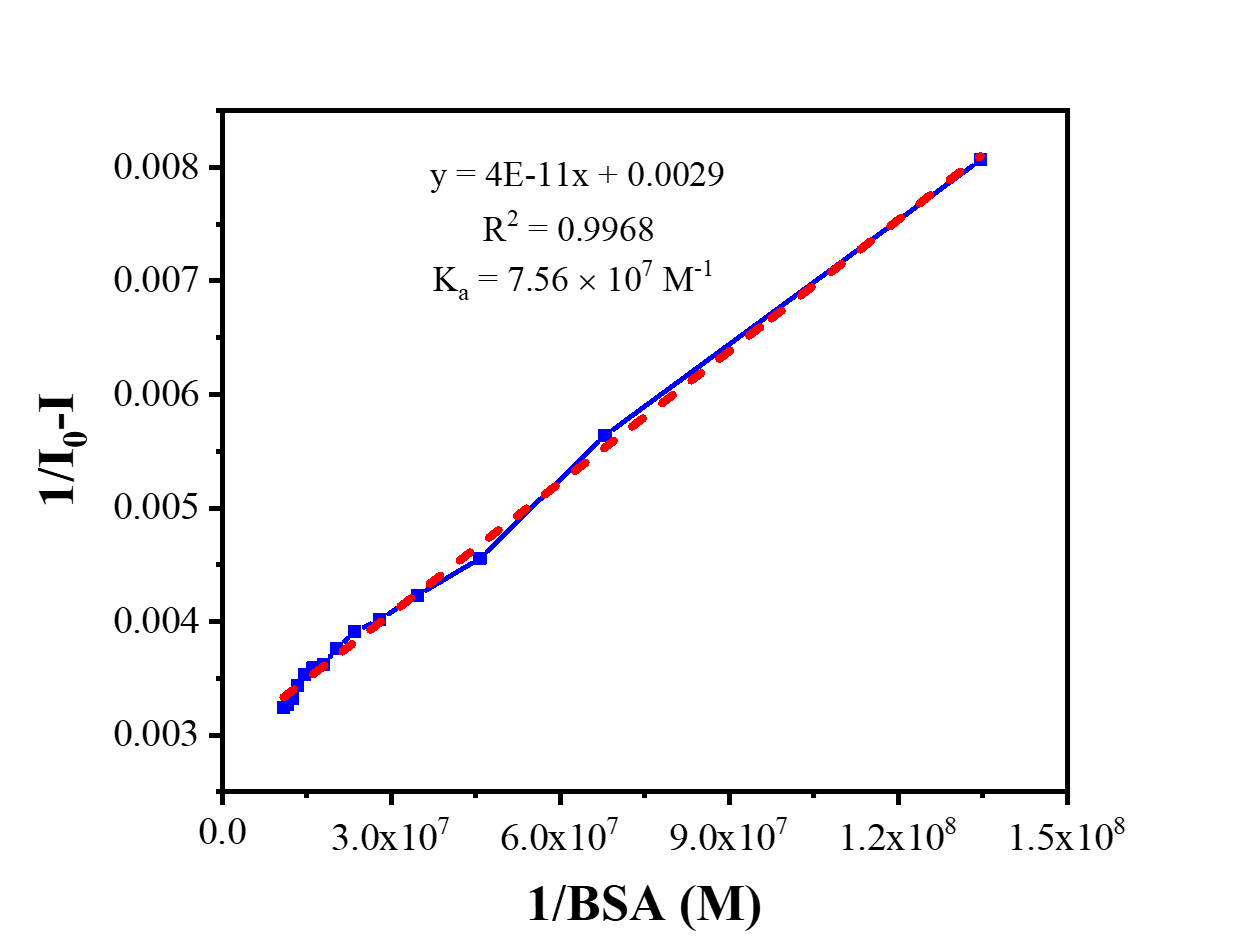


**Figure S14**. Benesi–Hildebrand plot of TPE-kana **1** with BSA in D/W.

**Stoke Shift Calculation:**

Calculated as difference between first absorption band in wavenumber and first emission band in wavenumber. The absorbance at 305.8 nm wavelength equal to 32701.11 cm^-1^ wavenumber and the emission at 476 nm wavelength equal to 21008.40 cm^-1^ wavenumber.

Stoke shift = 32701.11 cm^-1^  ̶ 21008.40 cm^-1^

=11692.71 cm^-1^


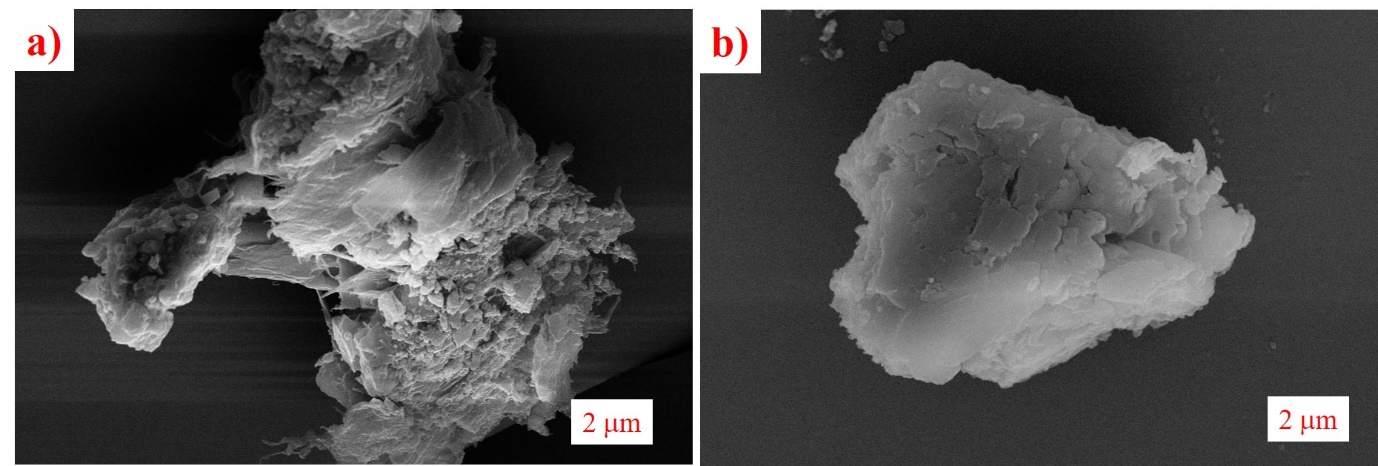


**Figure S15.** SEM images of TPE-kana **1** in the presence of BSA in water.

**References**

S1 Y. Xu, Q. Liu, X. Li, C. Wesdemiotis and Y. Pang, *Chem. Commun.*, 2012, **48**, 11313–11315.

S2 B. Liu, Y. Pang, R. Bouhenni, E. Duah, S. Paruchuri and L. McDonald, *Chem. Commun.*, 2015, **51**, 11060–11063.

S3 G. Dey, P. Gaur, R. Giri and S. Ghosh, *Chem. Commun.*, 2016, **52**, 1887–1890.

S4 F. Schlüter, K. Riehemann, N. S. Kehr, S. Quici, C. G. Daniliuc and F. Rizzo, *Chem. Commun.*, 2018, **54**, 642–645.

S5 G. Chakraborty, A. K. Ray, P. K. Singh and H. Pal, *Chem. Commun.*, 2018, **54**, 8383–8386.

S6 X. Xu, J. Huang, J. Li, J. Yan, J. Qin and Z. Li, *Chem. Commun.*, 2011, **47**, 12385–12387.

S7 L. Wang, L. Yang and D. Cao, *Sensors Actuators, B Chem.*, 2015, **221**, 155–166.

S8 Y. Suzuki and K. Yokoyama, *J. Am. Chem. Soc.*, 2005, **127**, 17799–17802.

S9 J. Bin Xiong, W. Z. Xie, J. P. Sun, J. H. Wang, Z. H. Zhu, H. T. Feng, D. Guo, H. Zhang and Y. S. Zheng, *J. Org. Chem.*, 2016, **81**, 3720–3726.

S10 F. Han, R. Zhang, Z. Zhang, J. Su and Z. Ni, *RSC Adv.*, 2016, **6**, 68178–68184.

S11 S. V. Nalage, S. V. Bhosale, S. K. Bhargava and S. V. Bhosale, *Tetrahedron Lett.*, 2012, **53**, 2864–2867.
