# Supplementary material for: Aminoglycoside antibiotic kanamycin functionalized tetraphenylethylene molecular probe for highly selective detection of bovine serum albumin protein
Source: Sci Rep. 2022 Jul 7;12:11526. doi: 10.1038/s41598-022-15890-4 (PMC9263133; doi:10.1038/s41598-022-15890-4)
Supplement: Supplementary file 2 — Supplementary Legends. [file 41598_2022_15890_MOESM2_ESM.docx]

**Supporting Information**

Provides further information about the characterization and sensing performance of TPE-kana **1**. The sensing performance, comparative detection limit, SEM images, UV-Vis absorption are available in supplesmentary information.
